# Energy stress activates AMPK to arrest mitochondria via phosphorylation of TRAK1

**DOI:** 10.1083/jcb.202501023

**Published:** 2026-01-30

**Authors:** Jill E. Falk, Tobias Henke, Sindhuja Gowrisankaran, Simone Wanderoy, Himanish Basu, Sinead Greally, Judith Steen, Thomas L. Schwarz

**Affiliations:** 1 F.M. Kirby Neurobiology Center, Boston Children’s Hospital, Boston, MA, USA; 2 Department of Neurobiology, Harvard Medical School, Boston, MA, USA; 3 Albert-Ludwigs-Universität Freiburg, Faculty of Medicine, Freiburg, Germany

## Abstract

Neuronal signaling requires large amounts of ATP, making neurons particularly sensitive to defects in energy homeostasis. Mitochondrial movement and energy production are therefore regulated to align local demands with mitochondrial output. Here, we report a pathway that arrests mitochondria in response to decreases in the ATP-to-AMP ratio, an indication that ATP consumption exceeds supply. In neurons and cell lines, low concentrations of the electron transport chain inhibitor antimycin A decrease the production of ATP and concomitantly arrest mitochondrial movement without triggering mitophagy. This arrest is accompanied by the accumulation of actin fibers adjacent to the mitochondria, which serve as an anchor that resists the associated motors. This arrest is mediated by activation of the energy-sensing kinase AMPK, which phosphorylates TRAK1. This mechanism likely helps maintain cellular energy homeostasis by anchoring energy-producing mitochondria in places where they are most needed.

## Teaser

Falk et al. identify a mechanism coupling mitochondrial movement to energy demand. When energy levels are low, the energy-sensing kinase AMPK phosphorylates the motor/adaptor protein TRAK1, arresting mitochondria via the actin cytoskeleton. This mechanism likely helps to position mitochondria where they are most needed.

## Introduction

Mitochondria are dynamic, energy-producing organelles that move, change shape, and tune metabolite production to match cellular demand. Cells such as neurons require a large amount of ATP for energy-intensive functions such as neurotransmission (Howarth et al., 2012; Attwell and Laughlin, 2001). In addition, neurons must employ mechanisms to distribute mitochondria throughout the entire cell, including its distal processes, to balance energy demand and supply. Thus, the movement and position of mitochondria are important for efficiently fueling cellular functions.

Processive mitochondrial movement occurs on microtubule tracks and requires dynein and kinesin for anterograde and retrograde movement. Mitochondria are coupled to these molecular motors via motor/adaptor proteins in which Miro and TRAK are central (van Spronsen et al., 2013; Glater et al., 2006). These motor/adaptors regulate mitochondrial position and movement, an example of which is the mitochondrial arrest caused by the O-GlcNAcylation of TRAK (Pekkurnaz et al., 2014). When shifted from a low- to high-glucose environment, mitochondria become immobilized by associating with the actin cytoskeleton. This arrest is caused by O-GlcNAcylation of TRAK1, which subsequently recruits to mitochondria the actin-interacting protein FHL2, thus immobilizing these organelles in regions of high glucose (Basu et al., 2021; Pekkurnaz et al., 2014). Another much-studied form of mitochondrial arrest is the response to rapid loss of mitochondrial membrane potential, including studies in which the electron transport chain (ETC) is inhibited by high concentrations of potent mitochondrial toxins (Wang et al., 2011; Shlevkov et al., 2016; Liu et al., 2012). Under these conditions, mitochondrial arrest is mediated by PINK1 and Parkin, and the degradation of Miro. The consequent arrest is a precursor to mitophagic elimination of the depolarized organelle (Jin et al., 2010).

While mitochondria are essential for all cell types, neurons are particularly vulnerable to fluctuations in fuel availability because they consume large amounts of ATP and yet are unable to store glycogen or perform beta oxidation (Yellen, 2018). The significance of mitochondrial energy production becomes evident from the many neurological diseases, both developmental and neurodegenerative, that are associated with mitochondrial dysfunction (Walker et al., 2022; Nunnari and Suomalainen, 2012; Ramos et al., 2021; Hou et al., 2019; Nicholls and Ferguson, 2013; Koopman et al., 2013). Notably, mutations in ETC genes required for mitochondrial energy production are the most common cause of pediatric metabolic disease, often with neurological consequences (Walker et al., 2022).

Here, we show that mitochondrial motility is sensitive to the ratio of ATP to AMP, a parameter that declines when ATP use is not adequately compensated by ATP production. AMP-activated protein kinase (AMPK), the ATP:AMP-sensing kinase, mediates this arrest by phosphorylating TRAK1 and triggering an association of the mitochondria with the actin cytoskeleton.

## Results

**Figure 1.**
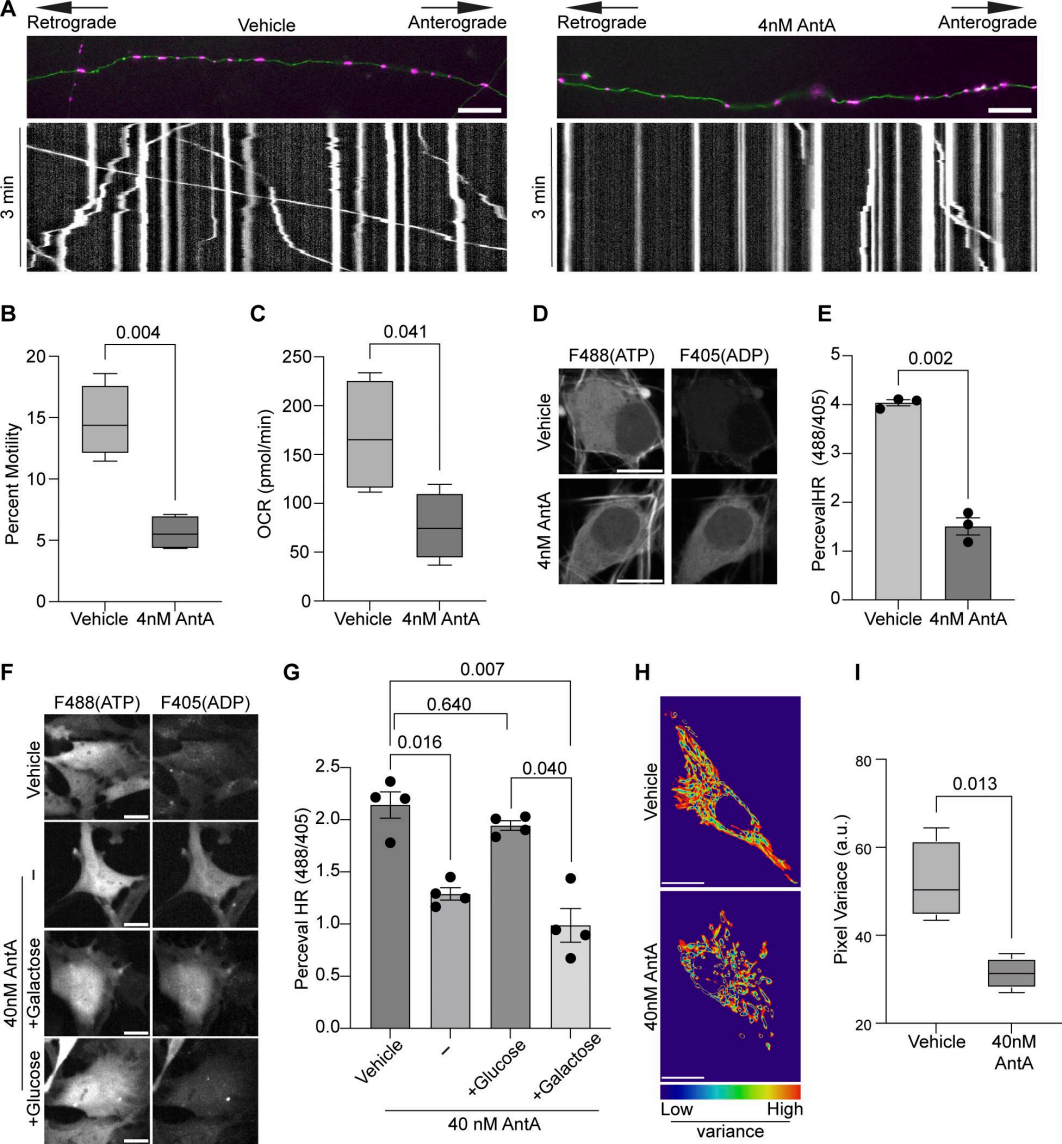
**ETC inhibition decreases the ATP:ADP ratio and arrests mitochondria. (A)** Kymographs of mitochondrial movement in cultured hippocampal neurons expressing mito-dsRED (magenta) and meGFP (green). Cells were treated for 1 h with either 4 nM AntA or vehicle and then imaged. **(B)** Quantification of mitochondrial motility from kymographs as in A. *n* = 9–13 axons per treatment from four independent animals. **(C)** Basal oxygen consumption (OCR) was measured in cortical neuron cultures 1 h after treatment with 4 nM AntA or vehicle. The consumption rate is normalized to cell number. *n* = 16–30 wells per condition from four independent animals. **(D)** Representative images of hippocampal neuron cell bodies expressing the fluorescent ATP:ADP ratio sensor PercevalHR. Cells were treated with 4 nM AntA or vehicle, as in A. **(E)** Quantification of fluorescence intensity ratio in hippocampal neurons expressing PercevalHR treated as in D. *n* = 11–19 cells per treatment from 3 independent animals. **(F)** Fibroblasts expressing PercevalHR were cultured in galactose media, treated for 1 h with 40 nM AntA or vehicle, and then imaged. In the lower two panels, 30 min after AntA addition, either glucose or galactose was added to the medium to a final concentration of 25 mM and immediately imaged. Glucose addition restored normal ATP/ADP ratios, but galactose addition did not. **(G)** Quantification of fibroblasts expressing PercevalHR treated as in F. *n* = 12–22 cells per condition from 4 independent transductions. **(H)** Representative image heatmaps show the degree of pixel variance as a reflection of mitochondrial movement. Fibroblasts expressing mito-dsRED, cultured in galactose media, were treated with vehicle or 40 nM AntA for 1 h. **(I)** Quantification of the variance in pixel intensity as an indicator of mitochondrial movement in fibroblasts treated as in H. *n* = 15–26 cells per condition from 4 independent animals. For all images, scale bars = 20 µm, bars on boxplots show the 10–90th percentile, and error bars on bar graphs show the SEM. P values for B, C, E, and I were calculated by two-tailed, unpaired *t* tests with Welch’s correction. For G, a one-way Welch ANOVA was performed with Dunnett’s T3 multiple comparison correction.

We examined the effects of 4 nM antimycin A (AntA), an inhibitor of the ETC complex III, on mitochondrial motility in rat hippocampal neurons expressing mitochondria-targeted dsRED. After 1 h, basal oxygen consumption was reduced, the cytoplasmic ATP:ADP ratio was reduced, and axonal mitochondria ceased moving (Fig. 1, A–E). We also tested rat embryonic fibroblasts (hereafter referred to as fibroblasts) to determine whether this effect was neuron-specific.

**Figure S1.**
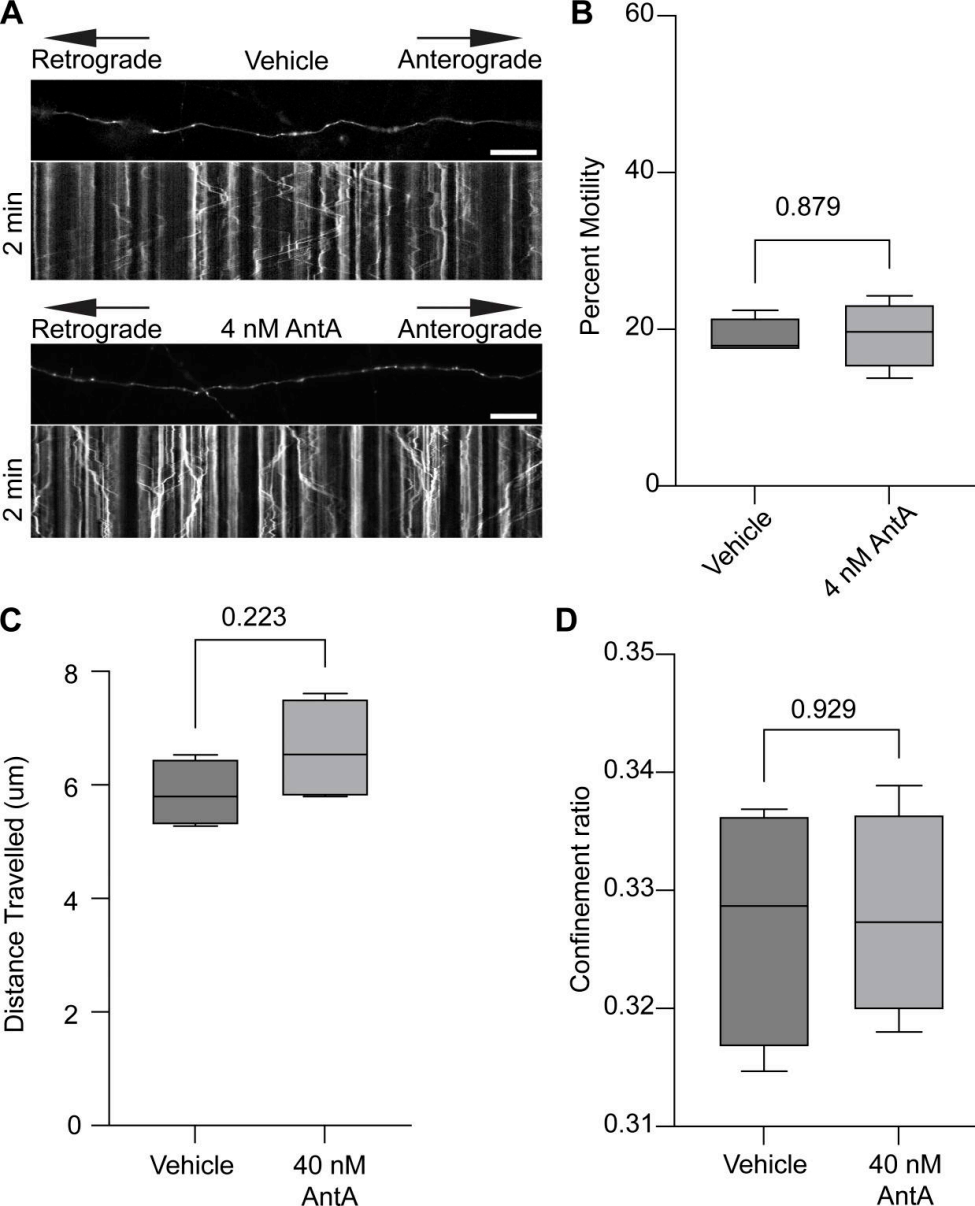
**Short treatments of** **AntA** **do not stop the movement of Rab5A-labeled endosomes or lysosomes. (A)** Kymograph of Rab5 motility in cultured hippocampal neurons expressing mEmerald-Rab5a and mCherry2. Cells were treated for 1 h with either 4 nM AntA or vehicle and then imaged. Imaging was performed in phenol red–free Hibernate E media to reduce background fluorescence. **(B)** Quantification of the Rab5 movement from axons as in Fig. 1 A. *n* = 8–10 axons per treatment from four independent animals. **(C)** Quantification of lysosome movement in fibroblasts. Cells were imaged live in galactose media after 1 h of treatment with vehicle or 40 nM AntA. LysoTracker Red DND-99 was used to visualize lysosomes. TrackMate 7 was used to track and quantify the total distance traveled by each lysosome track. **(D)** Quantification of the lysosomal confinement ratio from images acquired as in C. The confinement ratio of each lysosome track is defined as the net distance traveled divided by the total distance traveled. The net distance is defined as the distance between the starting and ending positions of the particle. The total distance traveled is the sum of the tracked segments. For all graphs, scale bars = 20 μm, and bars on boxplots show the 10–90th percentile. P values for B–D were calculated by two-tailed, unpaired *t* tests with Welch’s correction.

Standard cell culture techniques promote glycolytic growth in fibroblasts and most common cell lines, thus minimizing the effects of ETC inhibitors on ATP levels. To bypass this problem, we cultured fibroblasts in media containing galactose instead of glucose. In these conditions, inhibiting the ETC with 40 nM AntA for 1 h reduced the ATP:ADP ratio and the decline in the ratio could be rapidly reversed by supplying glucose 30 min after treatment (Fig. 1, F and G). Using software previously developed that measures pixel variance as an indicator of mitochondrial movement in nonneuronal cells (Basu and Schwarz, 2020), we found that 40 nM AntA also decreased mitochondrial motility in fibroblasts (Fig. 1, H and I). The arrest produced by the low concentrations of AntA used here is unlikely to be due to insufficient ATP because Rab5 and lysosomal movement was comparatively unaffected (Fig. S1, A–D).

**Figure S2.**
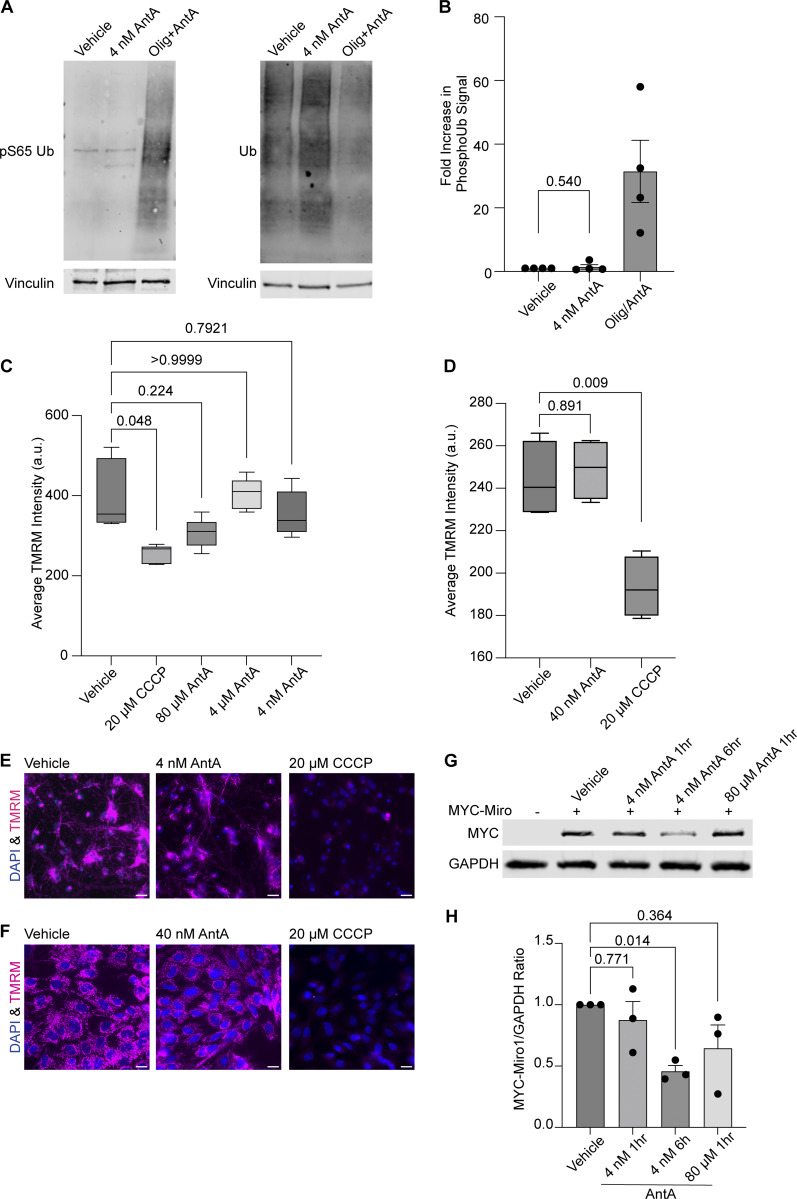
**4 nM AntA does not arrest mitochondria by the PINK1/Parkin pathway. (A)** Western blot of cortical neurons treated for 5 h with vehicle or 10 µM AntA and 1 µM oligomycin or 1 h of 4 nM AntA. Blots were probed for phospho-S65 ubiquitin or total ubiquitin. Anti-vinculin is shown as a loading control. **(B)** Quantification of phospho-S65 ubiquitin levels as in A; *n* = 4 biological repeats. **(C)** Quantification of mitochondrial membrane potential in hippocampal neurons stained with the reporter dye TMRM. Neurons were pretreated for 1 h with the indicated concentration of ETC inhibitor, after which TMRM and Hoechst were added. Cells were imaged live with both dyes and inhibitor present. Five cell fields were imaged from four independent biological repeats. TMRM intensity is normalized to cell count. **(D)** Quantification of TMRM intensity in fibroblasts grown in galactose media. Cells were pretreated for 1 h with the indicated concentration of ETC inhibitor, after which TMRM and Hoechst were added. Cells were imaged live with both dyes and indicated ETC inhibitors. 15–20 cell fields were imaged from 4 independent biological repeats. **(E)** Representative images from neurons treated with vehicle, 4 nM AntA, or 20 µM CCCP, as in C. **(F)** Representative images from fibroblasts treated with vehicle, 40 nM AntA, or 20 µM CCCP as in D. **(G)** Western blot of cortical neurons overexpressing MYC-Miro1 from the hSYN1 promoter. Cells were treated with AntA at the indicated concentrations and for the indicated times. Blots were probed with anti-MYC and with anti-GAPDH as a loading control. **(H)** Quantification of western blots as in G. *n* = lysates from three independent biological replicates. The P value for B was calculated by a two-tailed, unpaired *t* test with Welch’s correction. A one-way Welch ANOVA was performed with Dunnett’s T3 multiple comparison correction for C and D. P values were calculated by performing a blocked one-way ANOVA with Dunnett’s T3 multiple comparison correction for H. Scale bars = 20 μm. Source data are available for this figure: SourceData FS2.

The PINK1/Parkin pathway arrests depolarized mitochondria by modifying and then degrading Miro1 (Wang et al., 2011; Liu et al., 2012). To determine whether the concentrations of AntA used here caused mitochondrial arrest by the same pathway, we measured phosphorylated ubiquitin (pS65 ubiquitin), a well-established product of PINK1 activation (Koyano et al., 2014; Antico et al., 2021). In contrast to what is observed with higher concentrations of ETC inhibitors, 1 h of 4 nM AntA did not change the levels of pS65 ubiquitin (Fig. S2, A and B). This finding is consistent with the preservation of mitochondrial membrane potential as measured with tetramethylrhodamine methyl ester (TMRM) fluorescence in neurons and fibroblasts (Fig. S2, C–F). We also expressed myc-Miro1 from the neuron-specific human synapsin I promoter and found that the levels of myc-Miro1 were not significantly decreased by 1 h of exposure to 4 nM AntA (Fig. S2, G and H). With longer AntA treatments or higher concentrations, myc-Miro1 was degraded, consistent with previous findings (Wang et al., 2011; Liu et al., 2012).

**Figure 2.**
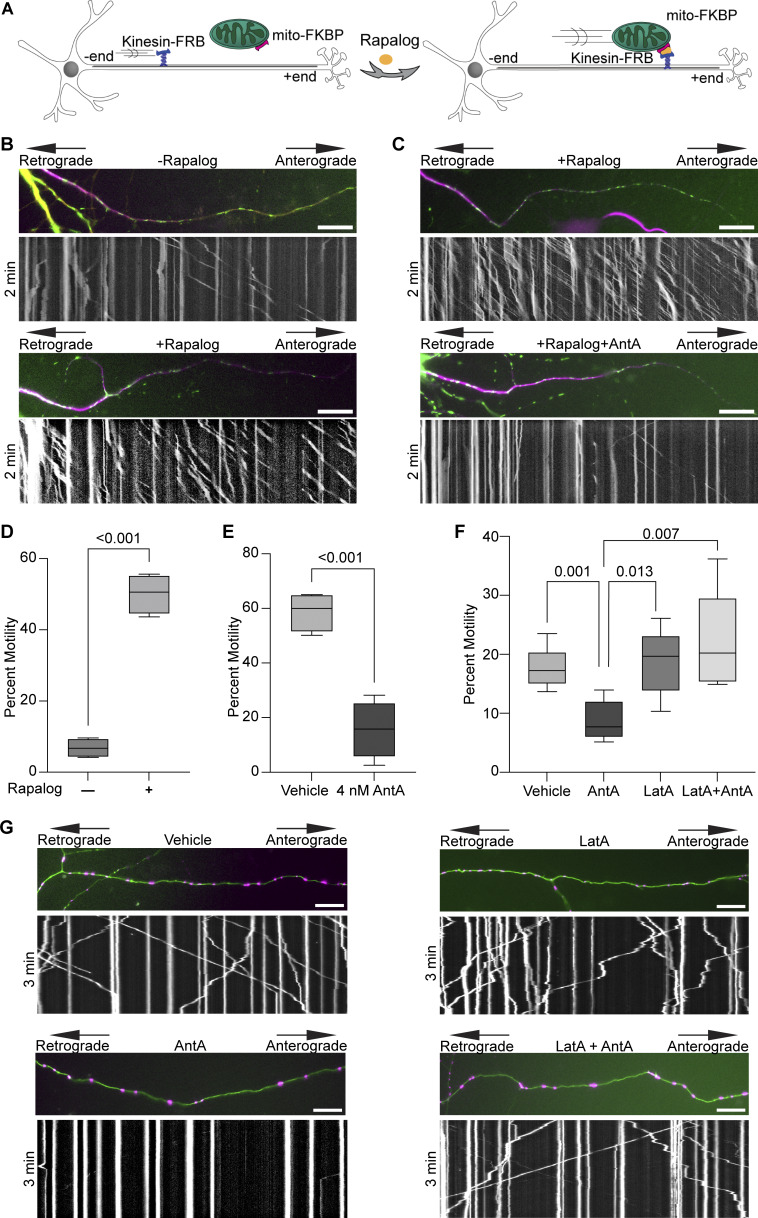
**ETC-inhibited mitochondria are arrested by the actin cytoskeleton. (A)** Cartoon of the rapalog system used to drive a constitutively active motor onto mitochondria. **(B)** Representative kymographs demonstrating the increased anterograde movement of mitochondria caused by the addition of rapalog. Hippocampal neurons expressing a mitochondria-targeted FKBP in green (mito-meGFP-FKBP) and a constitutive active kinesin (HA-Kif5b-MD-FRB) were co-expressed together with an axon initial segment marker TRIM46-mCherry in magenta (to identify axons) and iRFP706. Cells were treated with 1 µM rapalog or vector for 3–6 min and then imaged. **(C)** Representative kymographs demonstrating mitochondrial movement in neurons exposed for 1 h to 4 nM AntA or vehicle and then treated with rapalog as in B. **(D)** Quantification of mitochondrial movement with or without adding rapalog as described in B. *n* = 7–9 axons per treatment from four independent animals. **(E)** Quantification of rapalog-induced mitochondrial movement in hippocampal neurons treated with AntA or vehicle as described in C. *n* = 6–11 axons per treatment from four independent animals. **(F)** Quantification of mitochondrial motility in cultured hippocampal neurons treated for 3 h with 5 µM LatA or vehicle. After 3 h, 4 nM AntA or vehicle was added for one more hour. *n* = 7–10 axons per treatment from 6 independent animals. **(G)** Representative kymographs of mitochondrial movement from the experiment described in F. Mitochondria are magenta, and axons are green. For all graphs, bars on boxplots show the 10–90th percentile. P values for D and E were calculated by two-tailed, unpaired *t* tests with Welch’s correction. For all images, scale bars = 20 μm. For F, P values were calculated by performing a blocked one-way ANOVA with Tukey’s multiple comparison correction. Only P values ≤0.05 are shown.

**Figure S3.**
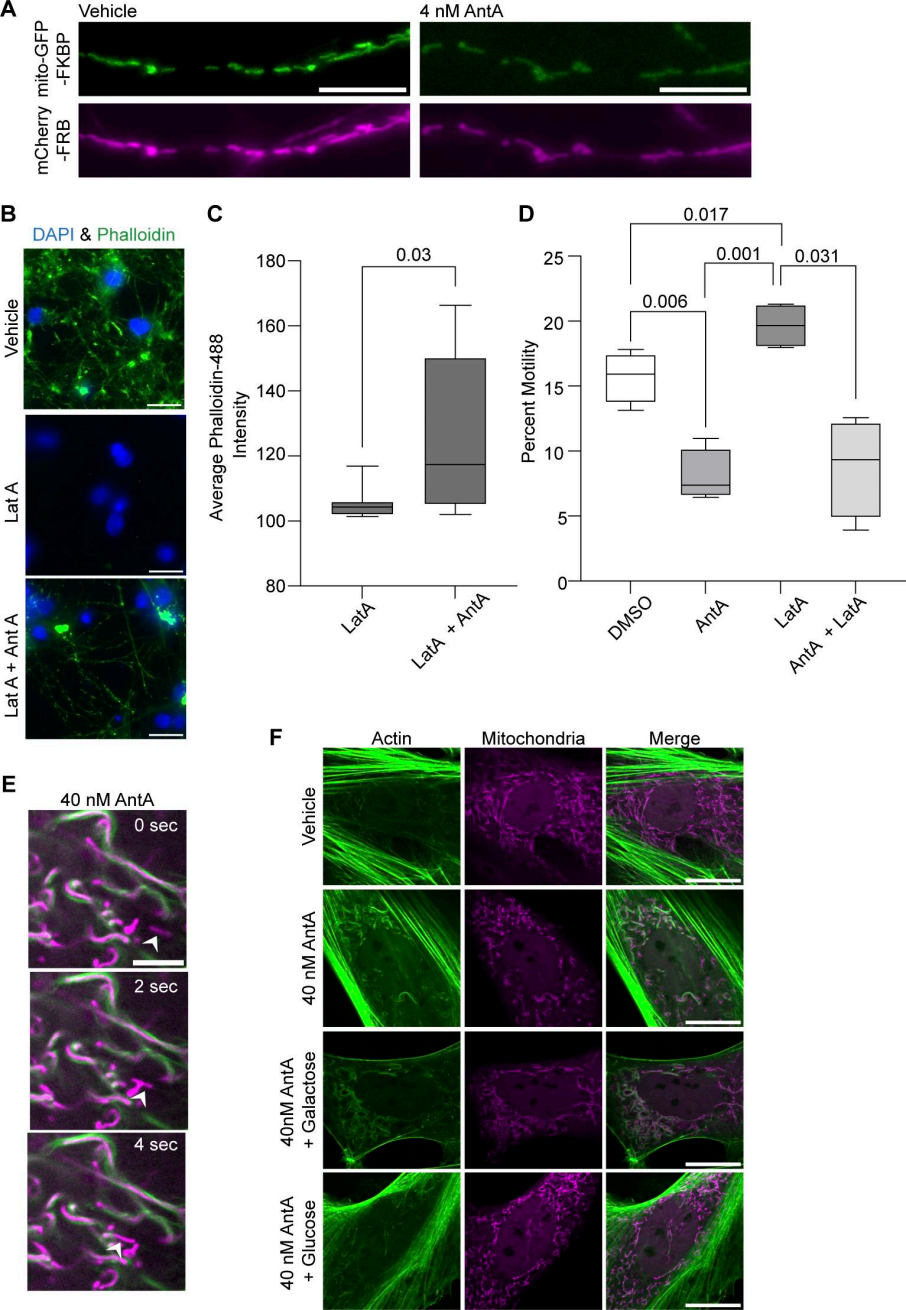
**Controls for rapalog motility experiments and perimitochondrial actin recruitment. (A)** Representative example neurites showing rapalog recruits mCherry-FRB to mitochondria in the presence of vehicle or AntA. Neurons were transfected with mito-GFP-FKBP and mCherry-FRB and pretreated for 1 h with either 4 nM AntA or vehicle before addition of 1 µM rapalog as in Fig. 2. **(B)** Representative images of hippocampal neurons that were fixed and stained with Alexa Fluor 488 phalloidin conjugate and Hoechst. Either control neurons or neurons pretreated with 4 nM AntA were exposed to 5 µM LatA. Note the persistence of a LatA-resistant pool of actin after pretreatment with AntA. **(C)** Quantification of filamentous actin from the average Alexa Fluor 488 phalloidin intensity of 60× imaging fields of neurons treated as in B. **(D)** Quantification of mitochondrial motility in hippocampal neurons pretreated for 1 h with 4 nM AntA and then 30 min with both 5 uM LatA and 4 nM AntA. Once arrested by treatment with AntA, LatA could not reverse the arrest. **(E)** Time-lapse series of fibroblasts treated for 1 h with 40 nM AntA and imaged as in Fig. 3 A. The arrowhead indicates a mitochondrion that appears to lack perimitochondrial actin and moves, while nearby mitochondria do not. **(F)** Representative images of rat embryonic fibroblasts transduced with GFP-F-tractin and mito-mRaspberry from the quantification in Fig. 3 D. Cells cultured in galactose were treated with 40 nM AntA or vehicle. One hour after drug treatment, cells either had glucose or galactose added to a concentration of 25 mM, or no further addition. Cells were imaged after 30 min. P values for C were calculated by two-tailed, unpaired *t* tests with Welch’s correction. A blocked one-way ANOVA was performed with Tukey’s multiple comparison correction for D. Select comparisons are shown for D. For supplemental images, 3A scale bars = 10 µm. The scale bar in 3E is 5 µm. For supplemental images, 3B and 3F scale bars = 20 μm.

There are two modes by which 4 nM AntA may be arresting axonal mitochondria: (1) they may be passively stopped, e.g., by being detached from their motors and microtubules as occurs upon Miro degradation; or (2) they may be actively anchored in place, e.g., by binding to microtubules or the actin cytoskeleton (Basu et al., 2021; Kang et al., 2008). To distinguish among these possibilities, we used a chemical-induced heterodimerization system to artificially tether a constitutively active kinesin to the mitochondrial outer membrane (Fig. 2 A) (van Spronsen et al., 2013). This system employs the FRB domain of mTOR, the FKBP domain of FKBP12, and rapalog (a metabolically inert rapamycin analog) to irreversibly couple two proteins of interest that are tagged with those domains. By targeting the FKBP domain to the outer mitochondrial membrane and attaching the FRB domain to the motor domain of human kinesin-1, the addition of rapalog forces these two fusion proteins to interact, thus driving active kinesin onto mitochondria (Kapitein et al., 2010). Passively arrested axonal mitochondria should then be dragged down the axons to microtubule + ends, but actively anchored mitochondria should be held in place if the anchor is robust enough to withstand the pulling force of the kinesin. While many mitochondria can be efficiently dragged down the axon in control conditions (Fig. 2, B and D), as previously observed (Gutnick et al., 2019), the mitochondria in AntA-treated cultures remained largely stationary (Fig. 2, C and E). This anchoring was not due to the inability of the kinesin to access the mitochondria; a mCherry-FRB reporter could be recruited to the mitochondria in the same conditions (Fig. S3 A). Thus, AntA-treated mitochondria behave as if they are actively held in place.

The actin cytoskeleton in several cell lines becomes transiently associated with mitochondria during mitochondrial depolarization (Fung et al., 2019; Fung et al., 2022; Chakrabarti et al., 2022; Li et al., 2015) and serves to immobilize mitochondria downstream of TRAK1 GlcNAcylation (Pekkurnaz et al., 2014; Basu et al., 2021). We therefore assessed whether the neuronal actin cytoskeleton was required for mitochondrial anchoring in response to AntA. Indeed, pretreatment with latrunculin A (LatA), which binds actin monomers to prevent their polymerization, prevented the arrest of axonal mitochondria by subsequent addition of AntA (Fig. 2, F and G). Oxidative stress has been shown to stabilize the neuronal actin cytoskeleton (Bernstein et al., 2006; Calabrese et al., 2022), and by quantifying phalloidin staining, we found that 4 nM AntA stabilized neuronal F-actin; LatA could not disrupt much of the already polymerized actin cytoskeleton once it had formed (Fig. S3, B and C). Consistent with that finding, while LatA pretreatment prevented mitochondrial arrest by AntA, mitochondrial movement could not be restored by adding LatA to cultures in which AntA had induced mitochondrial arrest and stabilized the actin (Fig. S3 D).

**Figure 3.**
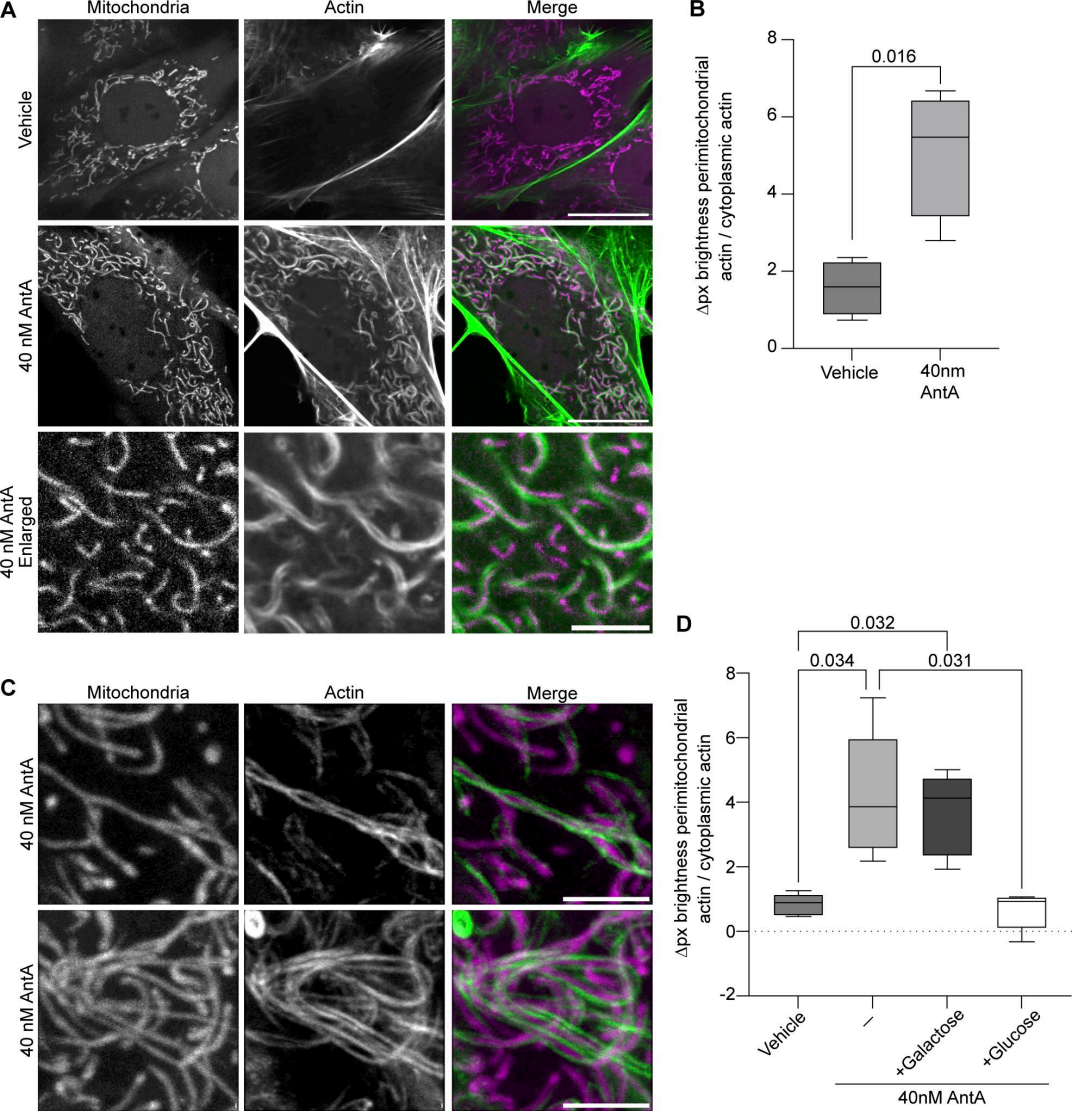
**Actin becomes stably associated with ETC-inhibited mitochondria. (A)**. Live-cell imaging of mitochondrial actin enrichment in fibroblasts treated for 1 h with 40 nM AntA or vehicle. Fibroblasts were cultured in galactose media to increase their dependence on ETC function. F-actin and mitochondria were visualized using GFP-F-tractin (green in merged image) and mito-mRaspberry (magenta in merged image). **(B)** Quantification of images treated as in A. *n* = 11–15 cells per condition from 4 independent animals. **(C)** Fibroblasts were transduced and treated with AntA as in A. Cells were imaged live on an Airyscan2 microscope. F-actin is shown as green, and mitochondria are magenta. **(D)** Fibroblasts transduced with GFP-Ftractin and mito-mRaspberry and cultured in galactose were treated with 40 nM AntA or vehicle as in A. One hour after drug treatment, cells either had glucose or galactose added to a concentration of 25 mM, or no further addition. Cells were imaged after 30 min. *n* = 10–11 cells per condition from 5 independent animals. For the top two rows of A, the scale bars are set to 20 μm; the bottom row of the same figure (enlargement) has scale bars set to 5 µm. The scale bar in C is 3 μm. The bars on boxplots show the 10–90th percentile. P values for B were calculated with two-tailed, unpaired *t* tests with Welch’s correction. For D, P values were calculated by performing a blocked one-way ANOVA with Tukey’s multiple comparison correction. Select P values are shown.

The dependence of the AntA effect on actin polymerization led us to evaluate whether this mild ETC inhibition induced mitochondria-associated actin structures. However, in fluorescence microscopy of axons, because of their small diameter, mitochondria and F-actin signals always overlap. The association of mitochondria with actin was therefore more easily quantified in fibroblasts (Fig. 3, A–D; and Fig. S3, E and F). Within 10–20 min after the addition of 40 nM AntA, actin filaments appeared to encase the mitochondria and mitochondria then remained in this close association with actin (Fig. 3, A and B). In contrast to earlier studies of a phenomenon termed “acute damage–induced actin” in which actin transiently associates with mitochondria (Fung et al., 2019; Fung et al., 2022; Li et al., 2015; Chakrabarti et al., 2022), the actin associations induced by 40 nM AntA remained for 12 h. When imaged at high resolution (Airyscan), actin fibers were visible that appeared to span the length of each mitochondrion, often with two filaments spiraling around the organelle (Fig. 3 C). These fibers resemble previous reports of perimitochondrial actin filaments (Fung et al., 2019; Fung et al., 2022; Chakrabarti et al., 2022). In rare cases, within an AntA-treated cell, a few mitochondria were observed that, for unknown reasons, did not detectably have adjacent actin; those mitochondria retained more motility than those associated with actin (Fig. S3 E). Consistent with these filaments forming in response to ATP depletion, adding glucose (but not galactose) quickly eliminated them (Fig. 3 D and Fig. S3 F).

**Figure 4.**
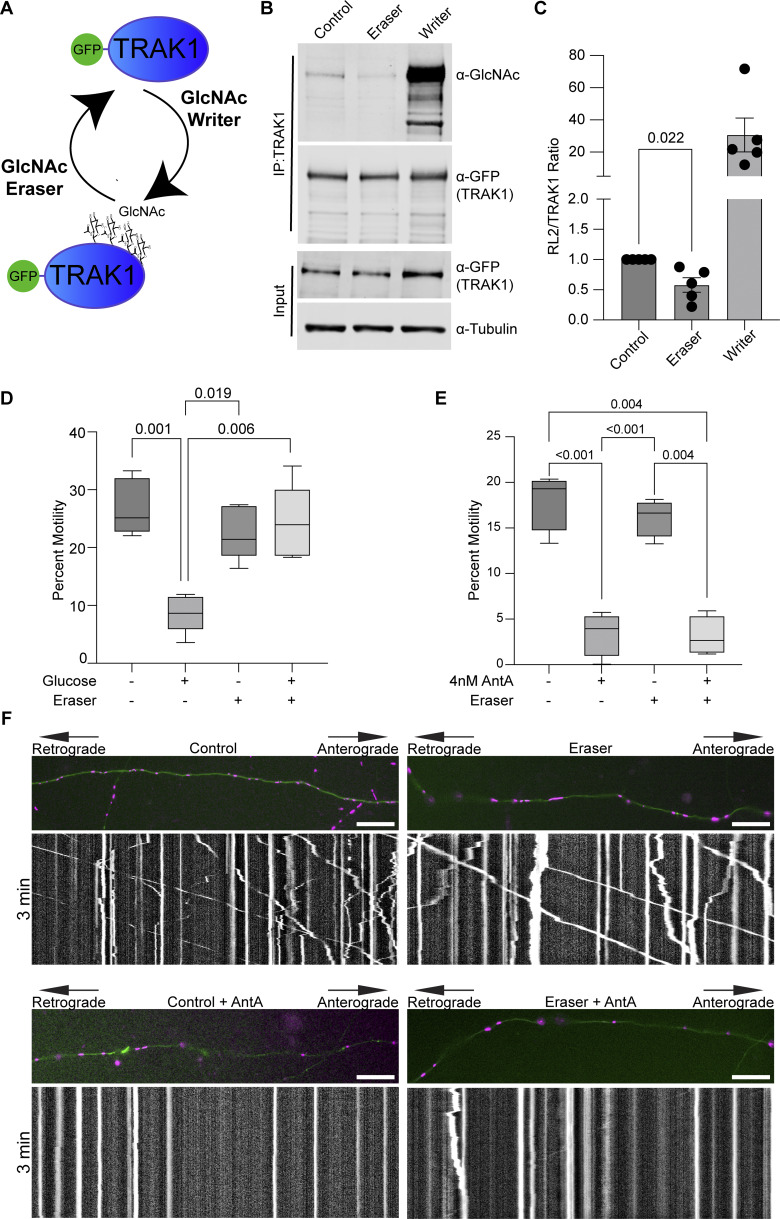
**De-GlcNAcylation of TRAK1 does not restore motility to AntA-arrested mitochondria. (A)** O-GlcNAc writer/eraser system consists of two engineered enzymes, an OGT and an O-GlcNAcase. Each enzyme is linked to a GFP nanobody that, when transfected into cells, can selectively target a GFP-tagged protein to add or remove O-GlcNAc. **(B)** O-GlcNAc writer and eraser can add and remove O-GlcNAc from GFP-tagged hTRAK1. GFP-hTRAK1 and the components of the O-GlcNAc writer or/eraser system were co-expressed in HEK293T cells. GFP-hTRAK1 was then immunoprecipitated using an anti-TRAK1 antibody. Immunoprecipitates and lysates were blotted and then probed with anti-RL2 (an antibody that recognizes O-GlcNAcylations), anti-GFP, and anti-tubulin. **(C)** Quantification of TRAK1 GlcNAcylation from blots as in B. *n* = 5 independent transfections per condition. **(D)** O-GlcNAC eraser prevents the mitochondrial arrest caused by 3 h of elevated glucose. Hippocampal neurons expressing GFP-hTRAK1, the O-GlcNAc eraser, mito-dsRED, and mNeonGreen were cultured in 5 mM glucose medium until DIV13. On DIV13, cultures were shifted for 3 h to 25 mM glucose medium or 25 mM sorbitol and then imaged for quantification of mitochondrial motility. *n* = 7–10 axons per treatment from five independent animals. **(E)** Quantification of mitochondrial motility after 4 nM antimycin treatment in hippocampal cultures expressing GFP-hTRAK1 and the O-GlcNAc eraser system. Cells were incubated for 2 h with 4 nM AntA, and imaging was done on DIV8–10. O-GlcNAc eraser did not prevent the AntA-induced arrest. *n* = 6–10 axons per treatment from four independent animals. **(F)** Example kymographs of mitochondrial movement in axons as in E. Mitochondria are magenta, and axons are green. For all images, scale bars = 20 μm, bars on boxplots show the 10–90th percentile, and error bars on bar graphs show the SEM. The P value for C was calculated by performing two-tailed, unpaired *t* tests with Welch’s correction. For D and E, P values were calculated by performing a blocked one-way ANOVA with Tukey’s multiple comparison correction. Only P values ≤0.05 are shown. Source data are available for this figure: SourceData F4.

**Figure S4.**
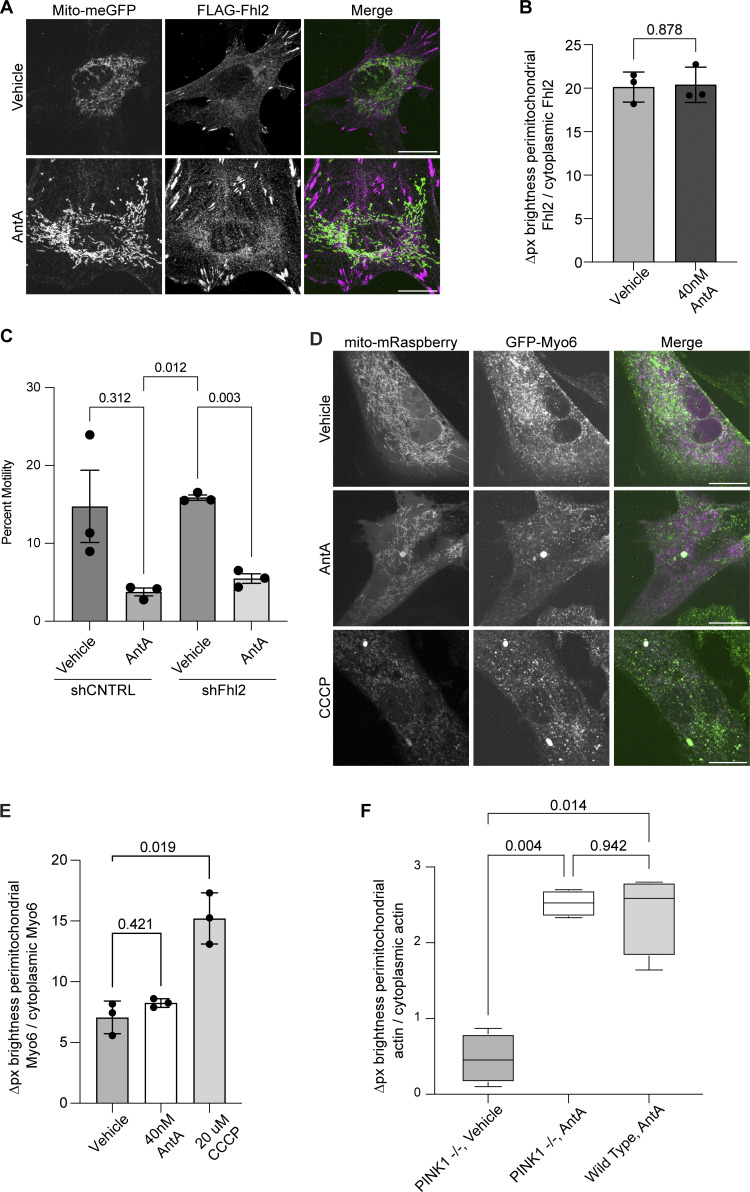
**Fhl2, Myo6, and PINK1 are not implicated in mitochondrial actin association after ETC inhibition. (A)** Anti-FLAG immunolocalization of FLAG-Fhl2 (magenta in merged image) in fibroblasts treated for 1 h with vehicle or 4 nM AntA. The FLAG-Fhl2 construct also expressed a P2A ribosome skip site followed by a mitochondria-targeted GFP (green in the merged image) to visualize mitochondria. **(B)** Quantification of images treated as in A. *n* = 10–12 cells per condition from 3 independent transductions. **(C)** Quantification of mitochondrial movement in cultured hippocampal neurons expressing mito-dsRED and meGFP and nontargeting control shRNA or a shRNA targeting Fhl2. Three days after transfection, cells were treated for 1 h with either 4 nM AntA or vehicle and then imaged. *n* = 7–9 axons per treatment from three independent animals. **(D)** Fluorescence microscopy of fibroblasts expressing GFP-Myo6 (green in merged image) and mito-mRaspberry to mark mitochondria (magenta in merged image). Cells were treated for 1 h with vehicle, 4 nM AntA, or 20 µM CCCP. **(E)** Quantification of images treated as in C. *n* = 10–23 cells per condition from 3 independent transductions. **(F)** Quantification of perimitochondrial actin enrichment comparing vehicle and AntA-treated wild-type and PINK1 knockout fibroblasts treated for 1 h with vehicle or 40 nM AntA. *n* = 10–12 cells from 4 independent transductions. The P value for B was calculated by a two-tailed, unpaired *t* test with Welch’s correction. An ANOVA with Dunnett’s T3 multiple comparison correction was performed for E. Blocked one-way ANOVAs were performed with Tukey’s multiple comparison correction for C and F. Select statistical comparisons are shown.

We previously described a mechanism by which mitochondria associated with and anchored to actin. This association was triggered by increasing O-GlcNAc transferase (OGT)–mediated GlcNAcylation of TRAK1 (Basu et al., 2021). To determine whether AntA employs the same mechanism, we used the writer/eraser system designed to tether either OGT or OGA (O-GlcNAcase) to a protein of interest (Ge et al., 2021; Ramirez et al., 2020). We co-expressed GFP-hTRAK1 with an empty vector control, a GFP nanobody coupled to OGT (O-GlcNAc writer), or a GFP nanobody coupled to OGA (the O-GlcNAc eraser) (Fig. 4 A) and asked whether the forced de-GlcNAcylation of TRAK1 prevented mitochondrial arrest by AntA. We first used HEK cells to test the efficacy of the OGT writer and OGA eraser constructs on co-expressed GFP-tagged hTRAK1. Upon immunoprecipitation (IP) of the TRAK1, the expected changes in the levels of O-GlcNAc moieties were observed (Fig. 4, B and C). Having confirmed the efficacy of the O-GlcNAc eraser for TRAK1 modification, we asked whether it could prevent the glucose-triggered arrest of neuronal mitochondria. As previously described (Pekkurnaz et al., 2014), we cultured hippocampal neurons in 5 mM glucose media until DIV13, at which point we added glucose to the culture to a final concentration of 25 mM for 3 h to induce GlcNAcylation of hTRAK1. Co-expression of the OGA eraser eliminated the OGT-triggered mitochondrial arrest (Fig. 4 D). In contrast, the OGA eraser did not rescue mitochondrial motility in AntA-treated cultures (Fig. 4, E and F), indicating that AntA acted through a different pathway. OGT drives actin association with mitochondria by recruiting the actin-binding protein FHL2 to TRAK1 (Basu et al., 2021). In response to AntA, however, no enrichment of FHL2 on mitochondria was observed (Fig. S4, A and B) and depleting Fhl2 in hippocampal neurons failed to rescue the AntA-induced mitochondrial arrest (Fig. S4 C). Mitochondrial depolarization by CCCP can cause actin association via the atypical myosin motor, MYO6 (Kruppa et al., 2018), but MYO6 colocalization with fibroblast mitochondria was not increased by 40 nM AntA (Fig. S4, D and E). Thus, neither GlcNAcylation of hTRAK1 nor recruitment of FHL2 or MYO6 appears responsible for arrest by 4 nM AntA. Furthermore, actin accumulated on mitochondria in PINK1 knockout fibroblasts in response to 40 nM AntA, confirming our findings (Fig. S2) that the arrest does not involve the PINK1/Parkin pathway (Fig. S4 F).

**Figure 5.**
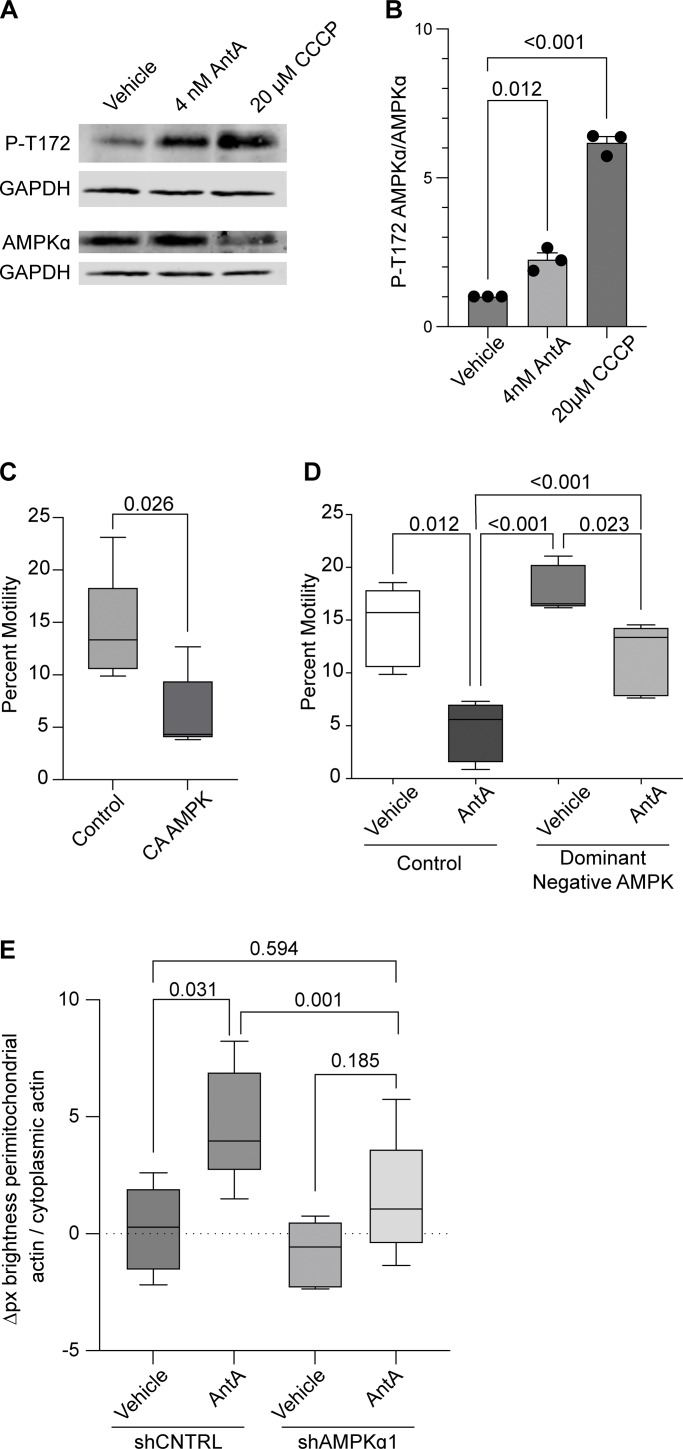
**AMPK is activated by ETC inhibition. (A)** Representative western blot of cortical neuron lysates was probed with an antibody specific for phospho-threonine 172 of AMPK and with anti-GAPDH. On a separate blot, the same lysates were probed with anti-AMPK and anti-GAPDH. Cultures were treated with ETC inhibitors for the indicated times and concentrations. **(B)** Quantification of replicate blots from cultures treated as described in A. n = lysates from three independent animals. **(C)** Quantification of mitochondrial motility in hippocampal cultures expressing constitutive active AMPK (CA-AMPK) or empty vector control, as well as mito-dsRED and meGFP. *n* = 7–10 axons per treatment from five independent animals. **(D)** Quantification of mitochondrial motility in hippocampal cultures expressing dominant negative AMPK (DN-AMPK) or empty vector control, as well as mito-dsRED and meGFP. DIV8–10 cultures were treated with 4 nM AntA or vehicle for 1 h and then imaged. *n* = 7–10 axons per treatment from five independent animals. **(E)** Quantification of the effects of a 4-day AMPK*α*1 knockdown on mitochondria-associated actin. Fibroblasts were cultured in galactose media to increase dependence on ETC function and then treated for 1 h with 40 nM AntA or vehicle. F-actin and mitochondria were visualized by live imaging using GFP-F-tractin and mito-mRaspberry and expressed either control or AMPK*α*1 shRNAs. shRNA constructs also expressed a nuclear-targeted iRFP (NLS-iRFP), and only cells expressing the NLS-iRFP signal were analyzed. *n* = 10–14 cells were imaged from 5 independent biological repeats. For all graphs, bars on boxplots show the 10–90th percentile, and error bars on bar graphs show the SEM. The P value for B was calculated by performing a blocked one-way ANOVA with Dunnett’s T3 multiple comparison correction. The P value for C was calculated by performing a two-tailed, unpaired *t* test with Welch’s correction. A blocked ANOVA was performed with Tukey’s multiple comparison correction for D and E. Only P values ≤0.05 are shown for D, and select comparisons are shown for E. Source data are available for this figure: SourceData F5.

**Figure S5.**
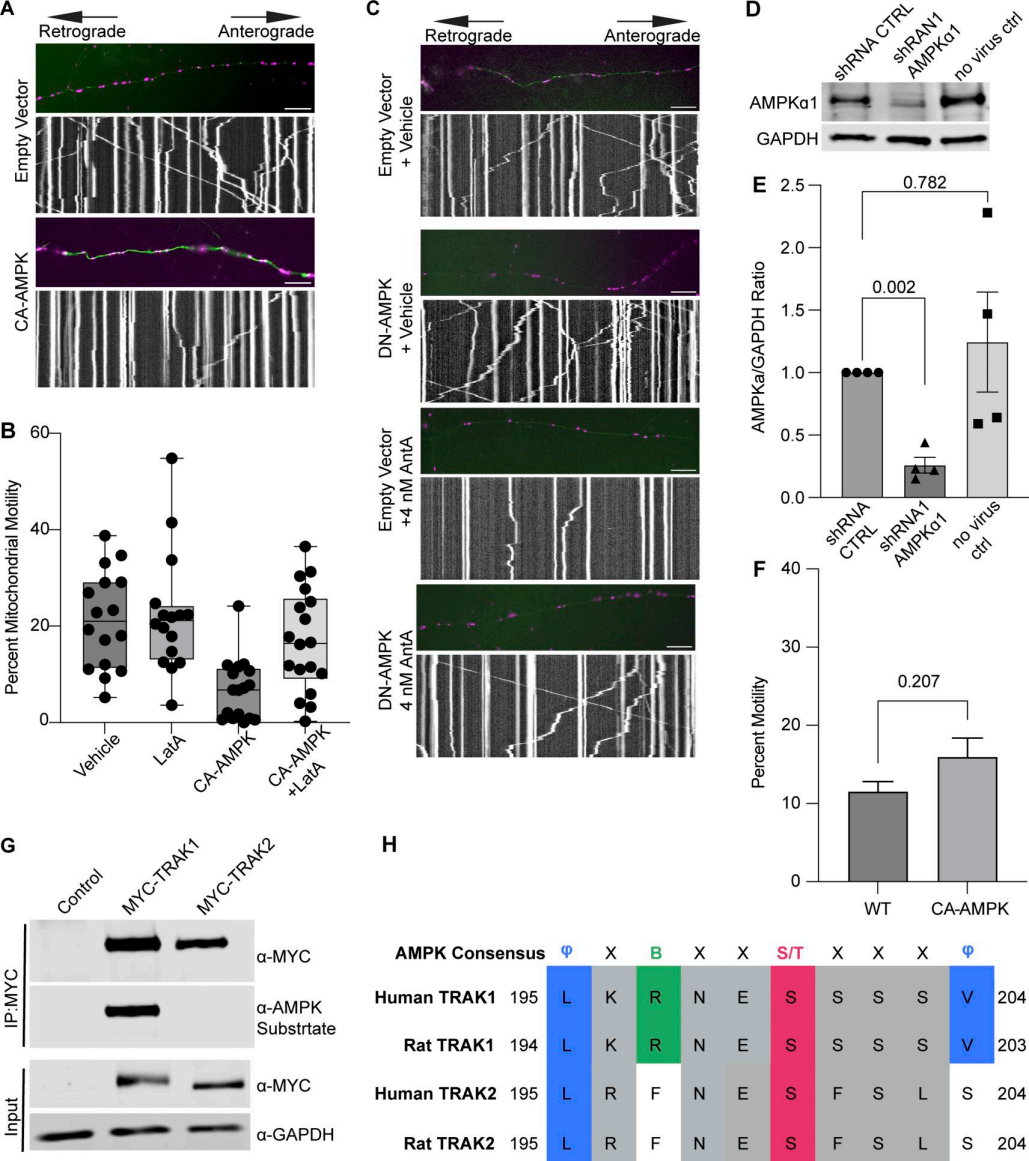
**Controls supporting a model in which AMPK regulates mitochondrial movement by phosphorylating TRAK1. (A)** Representative images and kymographs of data quantified in Fig. 5 C. Briefly, time-lapse images were acquired to measure mitochondrial motility in hippocampal cultures expressing constitutive active AMPK (CA-AMPK) or empty vector control, as well as mito-dsRED and meGFP. Mitochondria are magenta, and axons are green. **(B)** Quantification of mitochondrial motility in hippocampal cultures expressing constitutive active AMPK (CA-AMPK) or empty vector control. The cultures were treated for 4 h with 5 µM LatA or vehicle. *n* = 7–10 axons per treatment from 2 independent animals. The bars on each boxplot represent the min to max values. **(C)** Representative images and kymographs of data quantified in Fig. 5 D. Briefly, time-lapse images were acquired to measure mitochondrial motility in hippocampal cultures expressing dominant negative AMPK (DN-AMPK) or empty vector control, as well as mito-dsRED and meGFP. Neuron cultures were treated with 4 nM AntA or vehicle for 1 h and then imaged. Mitochondria are magenta, and axons are green. **(D)** Representative western blot of endogenous AMPK*α*1 in either untransduced fibroblasts or cells transduced with lentivirus expressing a shRNA targeting AMPK*α*1 or a nontargeting shRNA control. The blot was probed for AMPK*α*1 and GAPDH. **(E)** Quantification of AMPK*α*1 levels from blots as in D. *n* = 4 lysates from four independent animals. **(F)** Quantification of Rab5 motility in cultured hippocampal neurons expressing mEmerald-Rab5a, mCherry2, and either CA-AMPK or an empty vector control. Imaging was performed after 2 days of expression in Hibernate E media to reduce background fluorescence. *n* = 10 neurites per treatment from three independent animals. **(G)** HEK293T cells were cotransfected with CA-AMPK and MYC-tagged hTRAK1, hTRAK2, or an empty vector. MYC-hTRAK1 or MYC-hTRAK2 was then immunoprecipitated using an anti-MYC antibody. Immunoprecipitates and lysates were blotted and then probed for anti-phosphorylated AMPK substrate, anti-MYC, and anti-GAPDH immunoreactivity. **(H)** Comparison of the AMPK consensus sequence with the region surrounding S200 and S201 in human and rat TRAK1 and TRAK2. Amino acid properties of the consensus are as follows: Φ = hydrophobic; X = any amino acid; B = basic; S/T = phosphosite. Multiple sequence alignment was done with Clustal Omega and visualized in Jalview version 2 (Madeira et al., 2024; Waterhouse et al., 2009). UniProt and NCBI protein sequence identifiers are as follows: human TRAK1 = Q9UPV9, rat TRAK1 = NP_001128037.1, human TRAK2 = O60296, rat TRAK2 = Q8R2H7. A blocked one-way ANOVA with Dunnett’s T3 multiple comparison correction was performed for E. The P value for F was calculated by a two-tailed, unpaired *t* test with Welch’s correction. Source data are available for this figure: SourceData FS5.

Having excluded several previously described mitochondrial arrest mechanisms, we examined other potential means. Previous reports showing that mitochondria require their own ATP supply to remain motile suggested that AntA may arrest mitochondria by depleting ATP (Zala et al., 2013). At the concentrations used here, however, AntA did not decrease the movement of other organelles (Fig. S1); this mild ETC inhibition, though it altered the ATP/ADP ratio, cannot have caused neuronal ATP levels to fall below those necessary for motors to function. Similarly, if ATP depletion per se were responsible, LatA depletion of F-actin would not prevent the arrest. Inhibiting respiration or glycolysis, by shifting the ratio of ATP to AMP, can activate AMPK (Trefts and Shaw, 2021). This heterotrimeric kinase complex is expressed in neurons and can be directly activated by AMP, among other pathways (Lee et al., 2022; Tilokani et al., 2022; Toyama et al., 2016; Williams et al., 2011; Muraleedharan and Dasgupta, 2022; Lewis et al., 2018). Indeed, autophosphorylation at threonine 172 indicated that the kinase was activated in hippocampal cultures by 4 nM AntA within 1 h (Fig. 5, A and B). We therefore expressed in hippocampal neurons either a constitutively active form of the kinase (CA-AMPK) or a dominant negative form (DN-AMPK) (Egan et al., 2011; Mu et al., 2001). The overexpression of CA-AMPK decreased the fraction of moving neuronal mitochondria, mimicking the effect of AntA (Fig. 5 C and Fig. S5 A). The CA-AMPK–induced arrest could largely be rescued with LatA treatment (Fig. S5 B), and DN-AMPK largely prevented the mitochondrial arrest caused by AntA (Fig. 5 D and Fig. S5 C). Similarly, the actin associations required for the arrest in fibroblasts were largely eliminated by a shRNA targeting one of the catalytic subunits of AMPK (Fig. 5 E; and Fig. S5, D and E). Thus, AMPK activation is sufficient to arrest mitochondria and is necessary for the AntA-triggered, actin-mediated arrest. The AMPK-elicited mitochondrial arrest is most likely specific to mitochondria as Rab5 endosome movement was not inhibited by short AntA treatment or CA-AMPK expression (Fig. S1, A and B; and Fig. S5 F).

**Figure 6.**
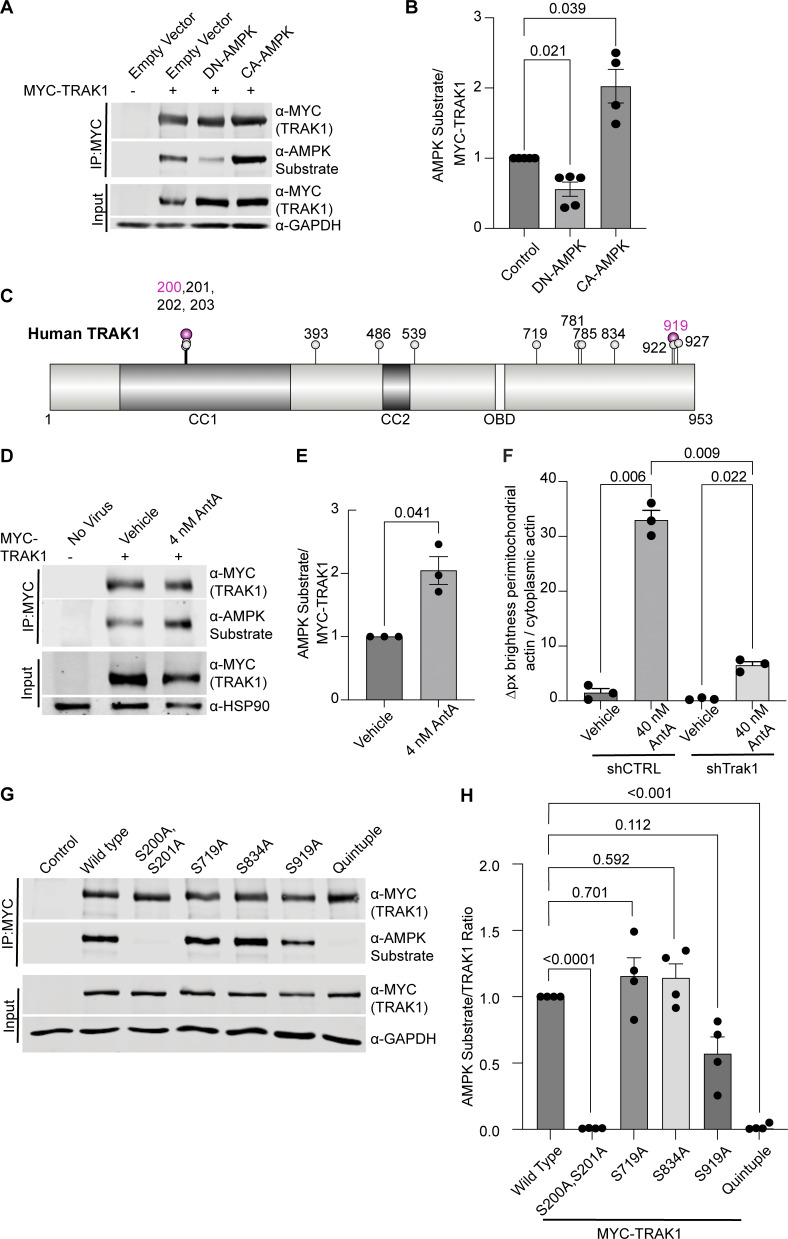
**AMPK phosphorylates TRAK1. (A)** MYC-tagged hTRAK1, as well as an empty vector, constitutive active AMPK (CA-AMPK), or dominant negative AMPK (DN-AMPK), was co-expressed in HEK293T cells. MYC-hTRAK1 was then immunoprecipitated using an anti-MYC antibody. Immunoprecipitates and lysates were blotted and then probed for phosphorylated AMPK substrate, anti-MYC, and GAPDH immunoreactivity. **(B)** Quantification of the phosphorylated AMPK substrate epitope normalized to anti-MYC levels from western blots as in A. *n* = 5 independent transfections per condition. Note that an outlier with a value of 9.5 AMPK substrate/MYC-TRAK signal was removed from the CA-AMPK treatment column, as well as the statistical analysis (this column thus contains 4 data points). **(C)** Schematic of the hTRAK1 phosphorylation sites. Mass spectrometry was performed on MYC-hTRAK1 expressed in HEK293T with CA-AMPK or DN-AMPK and then immunoprecipitated using an anti-MYC antibody. Immunoprecipitates were run on an acrylamide gel, and a ∼1 cm^2^ band of ∼100 KD was excised and used for mass spectrometry. Any site identified in the CA-AMPK samples is marked with a balloon. Magenta balloons are those that match the AMPK consensus sequence. The illustration was made with the IBS 2.0 online illustration tool using the Q9UPV9 UniProt human TRAK1 amino acid sequence (Xie et al., 2022). Predicted coiled-coil domains (CC1, CC2) and the OBD are indicated. **(D)** MYC-hTRAK1 was expressed from an hSYN1 promoter in cortical neuron cultures that were treated with 4 nM AntA or vehicle for 2 h. Anti-MYC immunoprecipitates and lysates were blotted and probed for phosphorylated AMPK substrate, MYC, and HSP90 immunoreactivity. **(E)** Quantification of TRAK phosphorylation in immunoprecipitates as in D. *n* = 3 cortical neuron cultures from three independent animals. **(F)** Quantification of perimitochondrial actin to assess the effects of a 3-day TRAK1 knockdown. Fibroblasts were cultured in galactose media to increase dependence on ETC function and then treated for 1 h with 40 nM AntA or vehicle. F-actin and mitochondria were visualized using GFP-F-tractin and mito-mRaspberry and expressed either nontargeting control or TRAK1 shRNAs. *N* = 11–17 cells per repeat from 3 independent animals **(G)** MYC-hTRAK1 with the indicated mutations of phosphorylation sites or with all 5 sites mutated (quintuple) was expressed in HEK293T cells co-expressing CA-AMPK. Anti-MYC immunoprecipitates and lysates were blotted and probed for phosphorylated AMPK substrate, MYC, and GAPDH immunoreactivity. **(H)** Quantification of G. *n* = 4 independent transfections per condition. For all graphs, bars on boxplots show the 10–90th percentile, and error bars on bar graphs show the SEM. The P values for B and H were calculated by performing a blocked one-way ANOVA with Dunnett’s T3 multiple comparison correction. The P values for F were calculated by performing a blocked one-way ANOVA with Tukey’s multiple comparison correction. Select P values are shown. P values for E were calculated by performing two-tailed, unpaired *t* tests with Welch’s correction. OBD, OGT-binding domain. Source data are available for this figure: SourceData F6.

To determine whether AMPK might regulate the motor/adaptor complex itself, we queried the ScanSite database for AMPK target consensus sites in the two human paralogs of each core component: Miro1/2 and TRAK1/2 (Obenauer et al., 2003). Multiple potential AMPK phosphorylation sites were predicted in TRAK1, and a single potential site was identified in TRAK2. We expressed myc-tagged TRAK1 in HEK293T cells either alone or together with CA-AMPK or DN-AMPK, immunoprecipitated the myc-tagged protein, and assessed its phosphorylation state with an antibody selective for the phosphorylated AMPK substrate motif (D’Amico et al., 2015; Wang et al., 2018; Zhang et al., 2016; Noren Hooten et al., 2016). Myc-TRAK1 was immunopositive for this phosphorylation in control conditions; the phosphorylation was decreased by the expression of DN-AMPK and increased by CA-AMPK (Fig. 6, A and B). We pursued the phosphorylation of TRAK1 by mass spectrometry of myc-TRAK1 immunoprecipitated from cells co-expressing CA-AMPK or DN-AMPK. Phosphopeptides were detected for each of the sites that had been predicted by ScanSite (Fig. 6 C). Importantly, when cortical neuron cultures were treated with 4 nM AntA, MYC-TRAK1 expressed from the human synapsin promoter became more immunopositive for the AMPK substrate motif (Fig. 6, D and E). If the modification of TRAK1 was essential for the AntA-induced, actin-dependent arrest of mitochondria, we reasoned that TRAK1 should be required for the accumulation of actin around mitochondria. As predicted, knockdown of TRAK1 by shRNA greatly reduced the formation of perimitochondrial actin (Fig. 6 F). Site-directed mutagenesis of five putative AMPK consensus sites eliminated the TRAK1 phosphorylation detected by the antibody against AMPK phosphosubstrates, and the vast majority of the signal was eliminated when only two sites, S200 and S201, were mutated to alanine (Fig. 6, G and H). The S200 and S201 consensus sites are not conserved in TRAK2, and myc-TRAK2 was not immunopositive for the AMPK phosphorylation site after expression with CA-AMPK (Fig. S5, G and H). TRAK1 has a putative AMPK phosphorylation site at S919, and the AMPK phosphosubstrate signal was marginally decreased by the S919A mutation. This site may also be phosphorylated by AMPK. Although TRAK2 has a predicted AMPK site at the equivalent position in its C terminus, TRAK2 was not immunopositive for the AMPK phosphosubstrate antibody. Thus, S200 and S201 are likely the primary AMPK phosphorylation sites of TRAK1.

**Figure 7.**
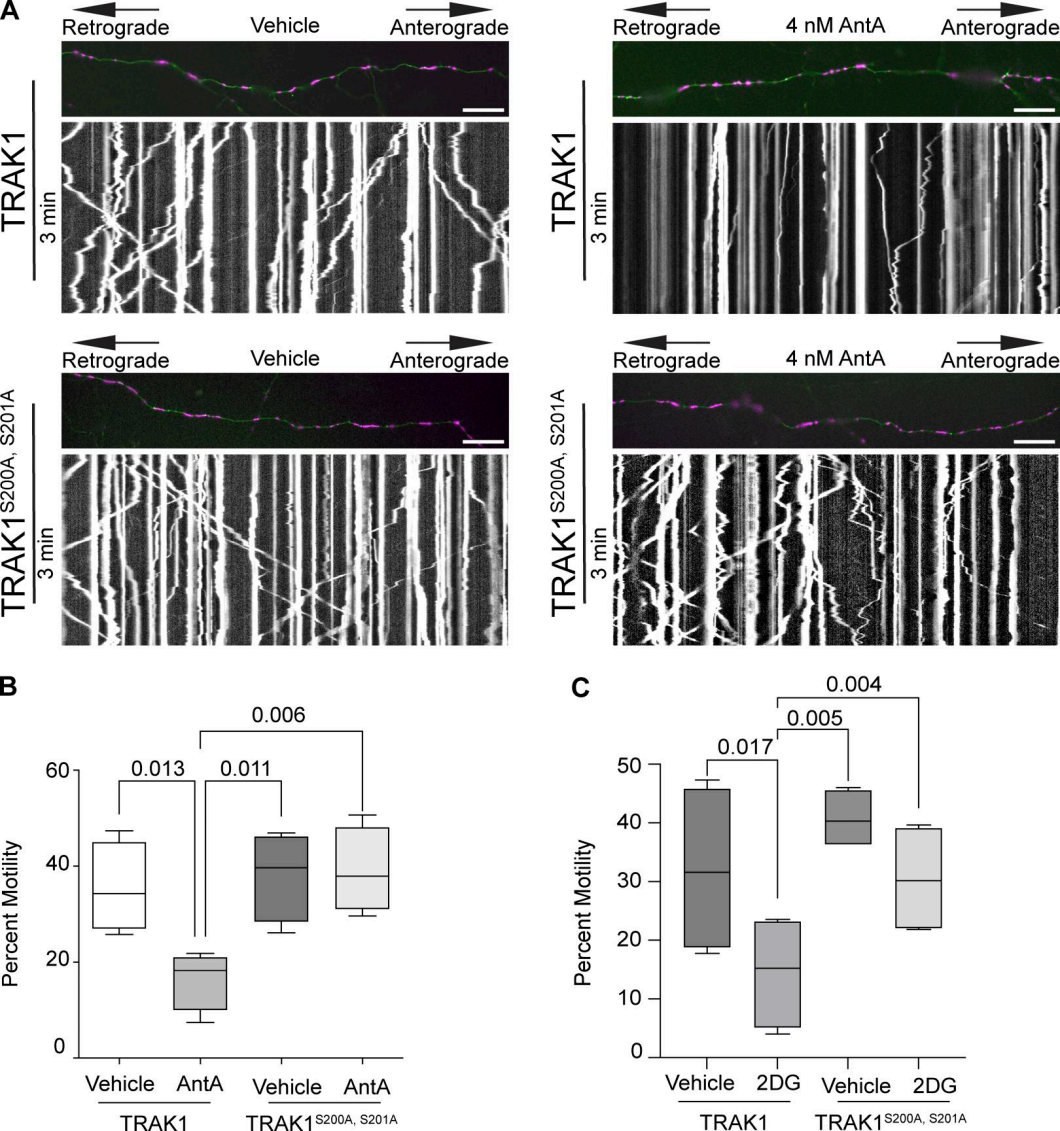
**Phosphorylation of S200 and S201 is necessary for mitochondrial arrest in response to energetic stress. (A)** Kymographs of hippocampal neurons expressing MYC-hTRAK1 or MYC-hTRAK1^S200A, S201A^, as well as mito-dsRED and meGFP. Cells were incubated for 2 h with 4 nM AntA or vehicle, and imaging was done on DIV9–11. Mitochondria are magenta, and axons are green. **(B)** Quantification of kymographs as in A. *n* = 6–9 axons per treatment from five independent animals. **(C)** Quantification of kymographs of mitochondrial motility in response to 1 h of 2-deoxyglucose treatment in hippocampal neurons expressing either MYC-hTRAK1 ^S200A, S201A^ or MYC-hTRAK1. 2-deoxyglucose treatment was done in neuron media lacking glucose and mitochondria, and neurites were visualized as in A. *n* = 6–9 axons per treatment from four independent animals. For all images, scale bars = 20 µm. For all graphs, bars on boxplots show the 10–90th percentile, and error bars on bar graphs show the SEM. P values for B and C were calculated by performing a blocked one-way ANOVA with Tukey’s multiple comparison correction. Select comparisons are shown.

We next asked whether the AMPK S200/S201 phosphorylation sites of TRAK1 altered mitochondrial movement after ETC inhibition. The expression of TRAK1 S200A/S201A alone did not significantly alter the mitochondrial movement in comparison with overexpression of wild-type TRAK1. However, after a 2-h treatment with 4 nM AntA, the overexpression of TRAK1 S200A/S201A completely eliminated the decrease in mitochondrial movement observed after the same treatment of neurons expressing wild-type TRAK1 (Fig. 7, A and B). As an alternative means of activating AMPK, we acutely inhibited glycolysis by replacing glucose with 2-deoxyglucose in the neuronal culture media, which caused the expected decrease in mitochondrial movement. This inhibition of motility was diminished by the overexpression of TRAK1 S200A/S201A, but not wild-type TRAK (Fig. 7 C).

## Discussion

Our experiments indicate that changes in the energetic state of a cell, as reflected by the ATP:AMP ratio, can feed back to regulate the movement of mitochondria, a major site of ATP production. Our findings support a model in which activation of AMPK causes the phosphorylation of the motor/adaptor protein TRAK1, which results in a change to the actin cytoskeleton that immobilizes mitochondria and may help position mitochondria at places where the ATP:AMP ratio is low. Evidence that this regulation is mediated by AMPK includes the following: (1) AMPK was activated by ETC inhibition as monitored by its phosphorylation state (Fig. 5, A and B); (2) CA-AMPK mimicked the effect of ETC inhibition (Fig. 5 C); (3) DN-AMPK prevented the arrest caused by ETC inhibition (Fig. 5 D); and (4) shRNA against AMPKa1 prevented the association of mitochondria with filamentous actin (Fig. 5 E), which is essential for the arrest (Fig. 2, F and G; and Fig. 3); (5) AMPK causes TRAK1 phosphorylation at two residues (S200 and S201) (Fig. 6); (6) shRNA against TRAK1 decreases the recruitment of actin to mitochondria in response to ETC inhibition (Fig. 6 F); and (7) mutation of the AMPK phosphorylation sites in TRAK1 prevents ETC inhibition from arresting mitochondrial motility (Fig. 7). Though the phosphorylation sites fit the consensus for AMPK targets, it remains possible that AMPK activates an intermediary kinase. The requirement for filamentous actin in this metabolic control of mitochondrial motility was shown by the ability of LatA to prevent the effect (Fig. 2) and the induction of actin filaments in the vicinity of mitochondria upon ETC inhibition (Fig. 3).

Metabolites, though often treated simply as substrates for cellular reactions, can also function as potent signaling molecules (Baker and Rutter, 2023; Chouchani, 2022). Activation of AMPK in response to energy stress is one of the best-characterized examples of such regulation. Axons, like all cells, contain many ATPases, including Na^+^/K^+^-ATPases, motor proteins, and actin itself. When mitochondrial production of ATP is impaired, these ATPases continue to generate ADP and AMP. When ATP consumption exceeds ATP resynthesis, AMP levels rise, and AMP binds to the gamma subunit of AMPK (Yeh et al., 1980). Activation of AMPK preserves energy homeostasis by enhancing pathways involved in energy conservation and inhibiting those that consume energy (Hardie, 2014; Auciello et al., 2014). AMPK activation inhibits growth-promoting pathways, upregulates autophagy, alters mitochondrial fission–fusion dynamics and flux through the TCA cycle, and activates mitochondrial biogenesis (Toyama et al., 2016; Egan et al., 2011; Malik et al., 2023; Di Nardo et al., 2014). The ATP:AMP ratio can vary greatly, even over short intracellular distances, allowing cells to optimize energy production where it is needed. One notable example of this is AMPK’s role in controlling mitochondrial localization to the leading edge of migrating cells (Cunniff et al., 2016), and TRAK1 phosphorylation by AMPK might contribute to this localization. Neurons are particularly sensitive to defects in energy distribution due to their large size, dynamic energy demands, and inability to store cellular fuels such as glycogen. Arresting mitochondria where ATP consumption is high, such as at synapses, can be a mechanism to match energy supply and demand.

Mitochondria also have many cellular functions outside of ATP production, which are reflected in the growing number of signaling pathways that control mitochondrial distribution. Through a related pathway, the GlcNAcylation of TRAK1 by OGT, mitochondria can be arrested when they encounter high glucose conditions (Pekkurnaz et al., 2014; Basu et al., 2021). Thus, both need for mitochondria’s output (ATP) and abundance of fuel such as glucose can have the similar effect of locally capturing mitochondria, and both achieve this in an actin-dependent manner that involves the posttranslational modification of TRAK (Basu et al., 2021; Pekkurnaz et al., 2014). These signals may operate at different time courses. A rise in AMP acts rapidly and is rapidly reversed if proper ATP synthesis is restored. A rise in glucose and the consequent change in TRAK1 GlcNAcylation may operate at a slower timescale. Perhaps this represents an acute need to correct inadequate ATP synthesis versus a chronic advantage to placing mitochondria where they can be most efficient. Other regulators of mitochondrial movement, and by extension position, include exposure to reactive oxygen species, increases in cytosolic calcium levels, and cell cycle progression (Chung et al., 2016; Debattisti et al., 2017; Wang and Schwarz, 2009). These pathways all impinge on the proteins of the motor/adaptor complex, making it a nexus for controlling mitochondrial positioning.

Several of these pathways act via TRAK1 and its paralog TRAK2, both of which are widely expressed. AMPK phosphorylation, however, was only readily detected on TRAK1 (Fig. S5 G). It is possible that TRAK2 is also an AMPK substrate whose phosphorylation sites are not recognized by the phosphosubstrate antibody (either at sites equivalent to S200 or S919). Nevertheless, because knockdown of TRAK1 (Fig. 6 F) greatly decreased the AntA-induced perimitochondrial actin, TRAK1 is the primary target to link actin to mitochondria during energetic stress in these cells. Few functional differences between these paralogs have been reported, but it has been proposed that TRAK1 preferentially localizes to axons and TRAK2 to dendrites due to differential affinities for kinesin versus dynein motors. This difference may arise from a conformational change in TRAK2 (not found in TRAK1) that prevents it from interacting with kinesin motors (van Spronsen et al., 2013). A similar mechanism was proposed for the *Drosophila* homolog Milton, whose splice isoforms differ in their interaction with kinesin (Glater et al., 2006). Studying how TRAK1 and TRAK2 differ in their regulation may provide insight into how mitochondria fulfill specialized functions in axons and dendrites.

TRAK1 is not the only mitochondrial substrate of AMPK; others regulate fission, local translation, the TCA cycle, mitophagy, and mitochondrial metabolism (Trefts and Shaw, 2021; Herzig and Shaw, 2018). Yet, little is known about how AMPK signaling is activated by different stimuli, particularly on mitochondria. Intriguingly, there appear to be distinct pools of AMPK subunits that preferentially localize to the mitochondrial outer membrane, raising the question of whether mitochondrial AMPK is selectively activated upon ETC inhibition or whether this also activates cytoplasmic and lysosomal AMPK-localized complexes (Schmitt et al., 2021, *Preprint*). Evaluating how different types of energy stress affect these targets may provide insight into how AMPK rewires metabolism. Because much work on AMPK has been conducted in cell lines, it may be particularly illuminating to find additional substrates in neurons where energy regulation is so critical.

AMP may not be the only signal activating AMPK when the ETC is inhibited. In neurons and other cells, increases in Ca^2+^ can activate CamKKβ, an upstream-activating kinase of AMPK. Supporting this hypothesis, synaptic activity has been shown to activate AMPK in neurons and arrest mitochondria at synapses (Li et al., 2020). Mild inhibition of the ETC might impair mitochondrial Ca^2+^ buffering and cause local Ca^2+^ increases that activate CamKKβ. Further work will need to evaluate the potential AMPK-activating role of Ca^2+^ and other potential signals after mild mitochondrial ETC inhibition.

Here, we find that mitochondria become anchored on the actin cytoskeleton in response to mild ETC inhibition; if we prevent the polymerization of actin with latrunculin, the arrest is prevented. Moreover, as observed in fibroblasts, each mitochondrion becomes tightly associated with one or more actin filaments (Fig. 3). Similar but exceptionally transient actin–mitochondrion associations have been reported to occur during the cell cycle and upon mitochondrial depolarization in nonneuronal cells (Fung et al., 2019, 2022; Li et al., 2015; Chakrabarti et al., 2022; Kruppa et al., 2018; Moore et al., 2016). We were unable to see whether ETC inhibition triggers a comparable enrichment of actin on axonal mitochondria due to the high density of actin in axons. Indeed, because actin filaments are already likely to be near most mitochondria in the close confines of the axon, it may suffice simply to attach them to those preexisting filaments.

What links actin to the outer membrane of ETC-inhibited mitochondria? ETC-inhibited mitochondria apparently are anchored independently of the actin-binding protein Fhl2 (Fig. S4, A–C) (Pekkurnaz et al., 2014; Basu et al., 2021). Formins such as INF2 or SPIRE1 are likely candidates that may activate local actin polymerization on mitochondria (Calabrese et al., 2022; Manor et al., 2015; Korobova et al., 2013). It is also tempting to speculate that in cases where damage to mitochondria compromises the ETC, actin recruitment may function to quarantine mitochondria and prevent them from fusing with their healthy counterparts. Actin filaments may also slow the process of mitophagy and allow time for ATP synthesis to be restored. Our data support a model in which actin filaments form an early response to mitochondrial malfunction, preceding mitochondrial depolarization, activation of PINK1, and recruitment of pro-mitophagy factors such as Parkin and MYO6. As has previously been proposed, mitochondrion–actin associations may also regulate the balance between oxidative phosphorylation and glycolysis to maintain ATP levels (Fung et al., 2019; Fung et al., 2022; Chakrabarti et al., 2022). In the case of neuronal stimulation, when ATP levels are low due to electrical activity, this arrest mechanism may help position mitochondria at active synapses. This arrest mechanism most likely promotes efficient ATP use and distribution and thereby supports the healthy function and survival of cells.

## Materials and methods

### Cell culture

Rat embryonic neurons: E18 hippocampal and cortical neurons were isolated from Long Evans rats as previously described (Nie and Sahin, 2012). Neurons were plated at a density of 5–7 × 10^4 cells/cm^2^. Neurons used for imaging were plated on 20 µg/ml polylysine and 4 µg/ml laminin (PDL)-coated 24-well or 35-mM glass-bottom dishes (P24-1.5H-N and D35-20-1.5-N, respectively; Cellvis). Neurons used for IPs or whole-cell lysates were plated on PDL-coated tissue culture-treated 10-cm or 6-well dishes. All cultures were grown in Neurobasal plus media (A3582901; Thermo Fisher Scientific) supplemented with L-glutamine and penicillin–streptomycin (10378-016; Thermo Fisher Scientific) and B27 supplement (17504044; Thermo Fisher Scientific) unless otherwise noted.

HEK293T/17 cells (CRL-11268; ATC) were cultured in DMEM (10566-016; Thermo Fisher Scientific) supplemented with penicillin–streptomycin (P4333; Thermo Fisher Scientific) and 10% FBS (S11150H; Atlanta Biologicals). HEK293T/17 cell stocks obtained from the ATCC were expanded and tested for *Mycoplasma* (on 6/25/2019) before use.

Rat embryonic fibroblasts: Fibroblasts were harvested from E18 Long Evans rat embryos (Charles River Laboratories) as previously described for mouse embryonic fibroblasts (Durkin et al., 2013). Cells were cultured in DMEM with standard supplements unless stated in the figure legend (see HEK293T/17 cell culture).

Procedures involving the use of animals followed NIH guidelines and were approved by the Institutional Animal Care and Use Committee at Boston Children’s Hospital (animal protocol numbers 00002228 and 00002539). Care was taken to minimize the number of animals used, as well as pain and suffering of the animals required for these procedures.

### Low-glucose and galactose cultures

Low-glucose neuron cultures were grown in standard Neurobasal plus culture media (see cell culture methods). On DIV5, Neurobasal plus media were exchanged for low-glucose neuronal media (Neurobasal A without glucose or pyruvate) (A2477501; Thermo Fisher Scientific) supplemented with 1X glutamine and penicillin–streptomycin (10378-016; Thermo Fisher Scientific), 1.2 mM sodium pyruvate (P8574; Sigma-Aldrich) 1 mM sodium L-lactate (71718; Sigma-Aldrich), 5 mM dextrose (G8270; Sigma-Aldrich), and 1X B27 supplement (17504044; Thermo Fisher Scientific). Neuronal cultures were fed daily (50% medium change) until DIV12, when motility experiments were performed.

Fibroblasts were seeded into 35-mm glass-bottom dishes at a density of 20,000–40,000 cells per dish in DMEM (see HEK293T/17 cell culture). The day after seeding, standard DMEM were fully exchanged for galactose media (glucose-free DMEM [1443001; Thermo Fisher Scientific], 10 mM galactose [G5388-100G; MilliporeSigma], 10% FBS, 2 mM GlutaMAX, 1 mM sodium pyruvate [P8574-5G; Sigma-Aldrich], and penicillin–streptomycin [P4333; Thermo Fisher Scientific]). Galactose media were fully exchanged each day, and experiments were performed after 2 days of growth in these media.

### Antibodies and reagents

Antibodies are listed in Table S3 and can be identified by their research resource IDs (RRIDs). All other reagents, such as plasmids and chemicals, are also listed, along with their source/vendor and catalog number, or, where appropriate, reference.

### Plasmid constructs

Table S1 along with Table S3 lists all primers and plasmids used in this study, as well as their respective sources. Apart from fluorescently tagged Sigma-Aldrich Mission shRNAs (available from Sigma-Aldrich), all plasmids created for this manuscript will be available on Addgene (https://www.addgene.org/Thomas_Schwarz/) or by request from the Schwarz lab. Details on the construction of plasmids made for this manuscript are listed below. All PCRs were performed with Q5 High Fidelity Master Mix; ligations were done with T4 ligase per the manufacturer’s protocol (M0202S; New England Biolabs), and ligation reactions were transformed into XL10-Gold *Escherichia coli* according to Mix and Go Competent Cell Kit (T3002; Zymo Research). Gibson assembly reactions were performed per manufacturer’s protocol (E2611L; New England Biolabs). Site-directed mutagenesis was performed with Q5 Site-Directed Mutagenesis Kit (E0552S; New England Biolabs). Clones were validated with Plasmidsaurus by whole-plasmid sequencing (https://www.plasmidsaurus.com).

### Published and purchased constructs

The following plasmids were previously published or were received as gifts: hTRAK2 (Davis et al., 2022; JF345), MYC-hTRAK1 (Pekkurnaz et al., 2014; JF190), HA-Kif5b-MD-FRB (Kapitein et al., 2010; JF184), nGFP-OGT-4 (Ramirez et al., 2020; JF426), (294) pcdna3.1-myc-OGA(1–400) (Ge et al., 2021; JF423), (369) pcdna3.1-HA-nLaGG-OGA-(544–706) (nGFP-C3) (Ge et al., 2021; JF425), MYC-Miro1 (Fransson et al., 2006; JF151), and PINK1-D110-YFP-FKBP (Lazarou et al., 2012; JF6). pLVX-CAG-MCS-IRES-mCherry was a gift from Matthew J. Lavoie (Brigham and Women’s Hospital, Boston, MA) (JF250), and pTomm20-mCherry-FKBP was a gift from Takanari Inoue (Johns Hopkins School of Medicine, Baltimore, MD) (JF41).

The following plasmids were obtained from Addgene: pMD2.G (CAT#12259; JF440), psPAX2 (CAT#12260; JF439), pAMPK alpha2 K45R (CAT#15992; JF451), CMV-PercevalHR (CAT#163061; JF639), pCherry-FRB (CAT#25920; JF196), pEBG-AMPK α1(1–312) (CAT#27632; JF450), FUGW-PercevalHR (CAT#49083; JF629), mEmerald-Rab5a-7(CAT#54243; JF124), mEGFP-N1 (CAT#54767; JF31), AmCyan-P2A-mCherry (CAT#45350; JF75), mRaspberry-Mito-7(CAT#55931; JF46), pEGFP-C1 F-tractin-EGFP (CAT#58473; JF258), pbetaActin-HALO-GFP-preActA (pERB254) (CAT#67762; JF11), paGFP-OMP25 (CAT#69598; JF19), pLV-EF1a-IRES-Hygro (CAT#85134; JF276), pLV-hSYN-RFP (CAT#22909; JF252), and dsRED2-Mito-7 (CAT#55838; JF262), piRFP702-N1 (CAT#45456; JF105), dsRED2-N1 (CAT#54493; JF30), pERB254 pbetaActin-HALO-GFP-preActA (CAT#67762; JF11), and meGFP-C1 (CAT# 54759; JF154).

pExpress-MYO6 was obtained from transOMIC (CAT# BC146764; JF155).

### Cloned constructs used for experiments

All primers and plasmids are listed in Tables S1 and S3. Plasmids for RNAi were obtained from Sigma-Aldrich, and the puromycin resistance cassettes were replaced with iRFP to generate shRNA AMPKa1 NLS-iRFP (JF587) and control shRNA2 NLS-iRFP (JF585). iRFP702 was amplified from plasmid JF105 with primers 551 and 738 (which also encoded the nuclear localization sequence, i.e., NLS). This PCR product and the shRNA vector backbone were digested with MluI-HF and BamHI-HF, and the products were ligated at a 3:1 ratio. A similar cloning strategy was taken to mark the TRAK1 shRNA (JF684) and control shRNA1 NLS-iRFP (JF306) with NLS-iRFP. NLS-iRFP was amplified from JF105 using primers 551 and 546 and cloned into the BamHI-HF and Kpn1-HF sites of the purchased shRNA vectors. 2X-FKBP-meGFP-OMP25 mts (JF188) was constructed as follows: FKBP was PCR-amplified from plasmid JF6 with primers 475 and 476. Plasmid JF61 and the FKBP PCR product were digested with AscI and Age1 and ligated at a 3:1 insert-to-vector ratio. To construct pLVX-CAG-mito-dsRED2 (JF264), mito-dsRED was amplified from JF262 with primers 519 and 518. This PCR product and JF250 were digested with BsrGI-HF and Not1-HF and ligated at a 3:1 ratio. To make iRFP-Trim46 (JF292), iRFP702 was amplified from JF105 with primers 552 and 553. This PCR and JF236 were digested overnight with MauBI and EcoRI-HF in the fast digest buffer from Thermo Fisher Scientific. Products were ligated at a 3:1 ratio. To make GFP-hTRAK1(JF474), JF154 was digested with BamHI-HF and EcoRI-HF. hTRAK1 was PCR-amplified from JF190 with primers 639 and 640 and digested with the same enzymes. The digested products were ligated at a 3:1 ratio. pLVX-EF1a-mito-mRaspberry (JF536) was made by PCR-amplifying mito-mRaspberry from plasmid JF46 with primers 705 and 706. Plasmid JF277 and the mito-mRaspberry PCR product were digested with NotI-HF and BsrGI-HF and ligated at a 3:1 insert-to-vector ratio. pLVX-CAG-F-tractin-GFP (JF529) was made by amplifying F-tractin-EGFP from plasmid JF258 using primers 696 and 695. Plasmid JF264 and the F-tractin-EGFP PCR product were digested with MluI-HF and BamHI-HF and ligated at a 3:1 insert-to-vector ratio. pLVX-CAG-meGFP-Myo6 (JF619) was made by amplifying meGFP-Myo6 from plasmid JF576 using primers 743 and 744. Plasmid JF580 and the meGFP-Myo6 PCR product were digested with SpeI-HF and MauBI and ligated at a 3:1 insert-to-vector ratio. pLVX-CAG-Fhl2-HA-P2A-mitomeGFP (JF626) was made by amplifying Fhl2-HA-P2A-mitomeGFP from plasmid JF570 using primers 741 and 742. Plasmid JF580 and the meGFP-Myo6 PCR product were digested with SpeI-HF and MluI and ligated at a 3:1 insert-to-vector ratio. pLVX-hSYN-MYC-Miro1 (JF525) was made by amplifying MYC-Miro1 from plasmid JF151 using primers 688 and 689. Plasmid JF276 and the MYC-Miro1 PCR product were digested with MluI-HF and EcoRI-HF and ligated at a 3:1 insert-to-vector ratio.

Site-directed mutagenesis was performed to make the TRAK1 phosphomutants. MYC-hTRAK1(S919A) (JF543) was made with JF190 as a template using primers 716 and 717. Likewise, MYC-hTRAK1(S719A) (JF557) was created by site-directed mutagenesis using plasmid JF190 and primers 723 and 724, MYC-hTRAK1(S200A, S201A) (JF559) using plasmid JF190 and primers 725 and 726, MYC-hTRAK1(T834A) (JF571) using plasmid JF190 and primers 732 and 733, and MYC-hTRAK1(S200A, S201A, S719A, T834A, S919A) (JF579) using plasmid JF565 with primers 732 and 733.

### Cloned constructs used as cloning intermediates/templates

pLVX-hSYN-mito-dsRED2 (JF276) was made by amplifying hSYN from JF252 using primers 530 and 531. The resulting PCR product and plasmid JF264 were digested with SpeI-HF and ClaI and then ligated at a 3:1 ratio. pLVX-EF1a-mito-dsRED2 (JF277) was made by amplifying the EF1a promoter from JF276 with primers 532 and 533. The PCR product and plasmid JF264 were digested with SpeI-HF and ClaI. Products were ligated at a 3:1 ratio. pLVX-CAG-mito-dsRED2 (JF264) was made by amplifying mito-dsRED from JF262 with primers 519 and 518. The resulting PCR product and JF250 were digested with BsrGI-HF and NotI-HF and cloned with T4 ligase at a 3:1 ratio. GFP-HALO-OMP25 (JF20) was made by amplifying the OMP25 mitochondrial targeting sequence from JF19 with primers 61 and 63. The vector plasmid JF11 and OMP25 insert were digested with EcoRI-HF and NotI-HF and ligated at a 1:1 ratio. To make pchBA-meGFP-HALO-OMP25(MTS) (JF61), Halo-meGFP was amplified out of JF51 with primers 218 and 217. Plasmid JF20 and the PCR product were digested with BsrGI and AscI and ligated at a 3:1 ratio. pLVX-CAG-MCS (JF580) was made by digesting plasmid JF264 and a gene fragment harboring a multiple cloning site (see primer table for sequence) with NotI-HF and MluI-HF. Products were ligated at a 3:1 ratio. hTRAK1(S200A, S201A, S719A, S919A) (JF565) was made by performing site-directed mutagenesis on plasmid JF560 using primers 725 and 726. hTRAK1(S719A, S919A) (JF560) was made by performing site-directed mutagenesis on plasmid JF560 using primers 725 and 726. pCMV-meGFP-Myo6 (JF576) was made by amplifying human Myo6 (GenBank: BC146764.1) from JF155 (pExpress-MYO6; transOMIC) using primers 731 and 728. Plasmid JF154 and the Myo6 PCR product were digested with EcoRI-HF and PspoMI and ligated at a 3:1 insert-to-vector ratio. To make TOMM20(MTS)-HaloTag-mEGFP (JF51), TOMM20(MTS)-HALO was amplified from JF47 using template primers 197 and 198. Plasmid JF31 and the PCR product were digested with HindIII-HF and BamHI-HF. Digest products were ligated at a 3:1 ratio. To make pCMV-TOMM20(MTS)-Halo-dsRED2 (JF47), JF39 was digested with EcoRI and BglII. The insert (TOMM20) was amplified from plasmid JF41 using primers 162 and 163. The products were ligated at a 3:1 ratio. pCMV-HALO-dsRED2-N1 (JF39) was made by amplifying the HALO tag from JF11 with primers 127 and 129. The PCR product and vector JF30 were digested with BamHI and EcoRI and ligated at a 1:1 ratio. pCMV-MCS-P2A-mCherry-N1(JF128) was made by amplifying P2A-mCherry from JF75 with primers 371 and 370. Plasmid JF31 and the P2A-mCherry product were digested with BAMHI-HF and NotI-HF and ligated at a 1:1 ratio. Fhl2-HA-P2A-meGFP(omp25 mts) (JF570) was made by PCR-amplifying Fhl2-HA-P2A from plasmid JF349 using primers 729 and 730. Plasmid JF146 and the Fhl2-HA-P2A PCR product were digested with BglII and PsPOMI and ligated at a 3:1 insert-to-vector ratio. To create pCMV-MCS-P2A-meGFP(omp25 mts) (JF146), JF128 was linearized by PCR with primers 415 and 418 (eliminating P2A-mCherry present in the parental vector). meGFP(omp25 mts) was amplified using primers 416 and 417 from JF61, and the products were assembled in a Gibson assembly reaction.

### Transfections and transductions

Neuron cultures were transfected on DIV5–9 with Lipofectamine 2000 as previously described (11668-019; Thermo Fisher Scientific) (Basu et al., 2021). Briefly, cells were gently washed three times with Neurobasal plus media lacking B27 (i.e., plain Neurobasal, A3582901; Thermo Fisher Scientific). A mix of endotoxin-free plasmid DNA, Lipofectamine 2000, and Neurobasal was added to the washed cells and incubated at 37°C. Cells were again washed twice with plain Neurobasal plus media and then cultured in standard neuron culture media at 37°C unless otherwise noted. Neuronal lentiviral transductions were done on DIV2–5 to minimize transduction of other cell types in the cultures. Lentivirus was thawed on ice and then added directly to the culture media, and the cells were returned to the incubator. The virus was washed off one to 2 days after transduction.

Transductions in fibroblasts were performed 1 day after plating, and the virus was washed off the following day.

Transfections in HEK293T cells were done at 70–95% confluence; HEK293T/17 cells were transfected with the desired plasmid constructs using PEI MAX (24765-100; Polysciences) or calcium phosphate (Kingston et al., 2003). One day after transfection, cells were washed and used for experiments one to 2 days later.

### Lentivirus production

HEK293T/17 cells (CRL-11268; ATC) were plated in 10-cm dishes and grown in standard DMEM (see Cell culture methods) until 95% confluent. Second-generation lentiviral packaging constructs were used in combination with various lentiviral transfer plasmids (listed in the Reagent Table) to make the desired virus. Viral packaging and transfer constructs were transfected into HEK293T/17 cells with PEI max (24765-100; Polysciences), and the mix was removed the following day. Virus was produced in these cells for 2 days, after which the supernatant was filtered through a 0.45-µm filter (97066-206; VWR), aliquoted and stored at −80, or concentrated to approximately ∼500×. To concentrate the virus, filtered viral supernatants were pelleted for 2 h at 50,000 × *g* in a Beckman Coulter Ultracentrifuge using an SW32Ti swinging bucket rotor, resuspended in phosphate-buffered saline, and snap-frozen in liquid nitrogen.

### Live-cell imaging

#### Motility analysis

All organelle movement experiments were performed on a widefield Nikon Ti Eclipse inverted microscope illuminated with SOLA LED light Engine (Lumencore) using a 63×, NA 1.4 Plan Apo objective (Nikon). Images were taken with a Zyla 5.5 sCMOS camera (Andor). Imaging was performed at 37°C in a humidified chamber supplied with a 5% CO_2_/air mix regulated by a Tokai Hit environmental chamber.

#### Imaging movement of mitochondria and Rab5 endosomes in neurons

Live-cell imaging of axonal mitochondria and axonal rab5 vesicles was performed on the widefield Ti-Eclipse microscope described above. Images were acquired at 0.5-s intervals for 2–5 min. Distal neurites were defined as axons if they exceeded 1 mm in length. Distal axonal mitochondria or Rab5 vesicles were imaged 2–3 days after transfection, ∼200 µM away from the axon tip. Rab5 vesicles were imaged in Hibernate E media (BrainBits HELF500) supplemented with B27 and penicillin–streptomycin (P4333; Thermo Fisher Scientific). Mitochondria were imaged in standard Neurobasal culture media unless otherwise noted. Kymographs were generated in Fiji using the Kymolyzer image analysis code (Schindelin et al., 2012). The data represented in each graph show the average percent motility as previously described with the exception of Fig. S5 B in which individual the percent motility for individual axons is shown (Basu et al., 2020; Basu et al., 2021). Briefly, mitochondrial tracks in each kymograph were manually traced and then analyzed in Fiji using the previously published Kymolyzer plug-in. Percent time in motion was scored for each mitochondrial track and then averaged for all tracks in the image.

Axonal rapalog imaging was performed the day after transfection, and mitochondria were imaged within 60–120 µm on the cell body. Axons were identified using Trim46-iRFP to mark the axon initial segment (Van Beuningen et al., 2015). Rapalog was added at a 1:500 dilution (10 µM) 3–6 min before imaging. Kymographs were generated and analyzed as described above.

#### Imaging mitochondrial and lysosomal movement in fibroblasts

Rat embryonic fibroblasts were used to image the movement of both mitochondria and lysosomes. Mitochondria were imaged on the widefield Ti-Eclipse microscope described above, and images were acquired at 0.5-s intervals for 3 min. Image analysis was performed using QuoVadoPro, which has been previously described in detail (Basu and Schwarz, 2020). Lysosomal motility was also imaged on the same widefield microscope, and these organelles were visualized by treating cells with 50 nM LysoTracker Red DND-99 for 30 min (Invitrogen, L7528). Image analysis was performed using TrackMate 7, a previously described Fiji plug-in (Ershov et al., 2022).

### Perimitochondrial actin imaging and quantification

Live-cell confocal actin imaging was done on either a Nikon Ti inverted microscope equipped with 405-, 488-, 561-, and 640-nm laser lines (Spinster) or one with 405-, 445-, 488-, 515-, 561-, and 640-nm laser lines (Garfunkel). Both microscopes use a Yokogawa W1 spinning disk with a 50-µm pinhole and a Zyla 4.2 Plus sCMOS camera (Andor). Imaging was performed with 60×/NA 1.4 Plan Apo objective (Nikon) using Nikon Elements Acquisition Software AR 5.02. Cells were maintained at 37°C with 5% CO2 with an OKO-Lab environmental chamber. Analysis to quantify perimitochondrial actin was performed in ImageJ as previously described^6^. In brief, intensity of the actin signal was quantified using a mask based on the mitochondrial marker but enlarged slightly so as to include actin immediately adjacent to the mitochondrion.

Super-resolution images were acquired on a Zeiss LSM 980 confocal microscope using the SR mode of an Airyscan detector. Images consisting of a 12-slice z-stack were collected (voxel size: 186 × 186 pixels, total field of view: 7.92 × 7.92 µm, z-step: 170 nm) using a Plan Apo 63×/1.4 Oil objective. The Airyscan detector was set at an 8-bit depth; digital gain, 1; binning, 1 × 1; line averaging, 2; and unidirectional scanning, scan speed 8. Image data were analyzed in ImageJ using the BioFormats plug-in.

### Mitochondrial membrane potential in neurons

As described above, neuronal cultures were grown on PDL-coated dishes in standard Neurobasal plus culture media. On the day of the experiment, cells were treated with drug or vehicle (as indicated in the figure legends), after which 100 nM TMRM (I34361; Thermo Fisher Scientific) was added according to the manufacturer’s protocol. 2 µM Hoechst (62249; Thermo Fisher Scientific) was also included in the same TMRM staining solution. Samples were imaged on the widefield Nikon Ti Eclipse microscope (as described above for neuronal motility analyses), and average field intensities were measured using one Z-section. Average TMRM intensities were normalized to the nuclear count per field (as measured by Hoechst staining).

### Mitochondrial membrane potential in fibroblasts

Fibroblasts were cultivated in galactose media for 2 days, at which point cells were treated with 40 nM AntA or vehicle. Cells were stained with TMRM and Hoechst as described above in the protocol for neurons. Image acquisition was performed using a Nikon Ti Eclipse widefield microscope (as described above for neuronal motility analyses). Analysis was performed by measuring the mean field brightness in both TMRM and Hoechst channels. The TMRM signal was normalized to the average Hoechst signal.

### PercevalHR

Ratiometric PercevalHR imaging in neuronal cell bodies was performed on the spinning disk Garfunkel (described in the section on live-cell actin imaging). Neurons were transduced with lentivirus harboring the PercevalHR sensor (Tantama et al., 2013) transduced on DIV2 and then imaged between DIV7–9. Ratiometric images were taken by acquiring Z-stacks with a Plan Apo 100× Oil objective and a Zyla 4.2 Plus sCMOS camera (Andor). Cells were illuminated with the 488 and 405 lasers, and emission spectra for both channels were collected using the 525/36 filter set. Seventeen Z-sections were taken for each field at 0.5 µm per section. A summed intensity projection was made for each image, and mean plate background intensities were calculated and subtracted from the PercevalHR signal in each channel. The ratio of the ATP (488) to ADP (405) summed cellular signal was then calculated. Imaging was performed in Hibernate E media (BrainBits HELF500) supplemented with B27, and cells were maintained at 37°C with an OKO-Lab environmental chamber. Imaging PercevalHR in fibroblasts was performed similarly after transduced cells had been cultured in galactose media for 2 days. Fibroblasts were imaged with a 20× objective and nine 1-µm sections and were maintained while imaging at 37°C with 5% CO_2_ with an OKO-Lab environmental chamber.

### Imaging fixed cells

#### Neuronal phalloidin staining

Neurons were fixed for 5 min at room temperature in a formaldehyde solution (3.5% paraformaldehyde [15714; Electron microscopy science], 4% sucrose [S0389-1KG; Sigma-Aldrich] in 1X dPBS, pH 7.1 [i.e., dPBS, 14080055; Thermo Fisher Scientific]), and then immediately washed three times in dPBS. Cells were permeabilized for 15 min with 0.3% Triton X-100 (T8787-250ML; Sigma-Aldrich) dissolved in dPBS. The permeabilization buffer was then washed once with dPBS and then phalloidin buffer (1% BSA [wt/vol] dissolved in dPBS). The phalloidin buffer was washed off, and the sample was incubated for 30 min with AF488-conjugated phalloidin (sc-36379; Santa Cruz) dissolved in 1:1,000 in phalloidin buffer. Images were acquired on the widefield Nikon Ti Eclipse described for neuronal motility analyses. Imaging was done at 25°C.

#### Myo6

Lentiviruses expressing Myo6-meGFP and mRaspberry-Mito-7 were transduced into fibroblasts grown in standard DMEM, which was exchanged for galactose media upon the addition of the virus. The lentivirus was washed off after 1 day, and cells were treated with AntA or vehicle after 2 days in galactose media. Fibroblasts to be treated with CCCP were transduced and treated in standard DMEM. Three days after plating, drug-treated fibroblasts were fixed for 5 min in formaldehyde buffer (3.5% paraformaldehyde, 4% sucrose dissolved in 1X dPBS, pH 7.1) prewarmed to 37°C. The formaldehyde buffer was washed off with 1X dPBS, pH 7.1, at room temperature. Fixed cells were imaged on the spinning disk described above (Garfunkel). Imaging was done at 25°C.

#### Immunocytochemistry

Cells were fixed at 37°C for 5 min in prewarmed formaldehyde buffer (3.5% paraformaldehyde, 4% sucrose dissolved in 1X dPBS, pH 7.1). The formaldehyde solution was washed off with dPBS at room temperature, and cells were permeabilized for 15 min in 0.3% Triton X-100 (T8787-250ML; Sigma-Aldrich) diluted in dPBS. Samples were washed three times in dPBS and incubated at 4°C overnight in blocking buffer (5% BSA wt/vol dissolved in dPBS). For Fhl2-HA-P2A-mitomeGFP, a CoraLite Plus fluorescent secondary–coupled anti-HA primary antibody (CL647-66006; Proteintech) diluted 1:200 in antibody buffer was added to the samples for 2 h, after which samples were washed three times with dPBS and imaged. Fixed cells were imaged on the spinning disk described above (Garfunkel). Imaging was done at 25°C.

#### Immunoprecipitations

HEK293T/17 cell IPs: Two to 3 days after transfection, HEK293T/17 cells were washed once with PBS and then lysed on ice with 1.8 mL NP-40 lysis buffer (1% NP-40, 15 mM Tris-HCl, pH 7.5, 150 mM NaCl, 1 mM EDTA, pH 8.0, 1 mM dithiothreitol (DTT), 1 mM PMSF, and a protease inhibitor cocktail (11836170001; Roche)). Lysates were centrifuged at 13,000 × *g* in a tabletop centrifuge for 10 min at 4°C. Supernatants were collected, 50uL of which as boiled with SDS Laemmli buffer and used as input samples. The remaining supernatant was used for the IP.

For IP of MYC-TRAK2 and MYC-TRAK1 (wild-type and phosphomutants), 3 µg of anti-MYC (SC-40; Santa Cruz) was added to lysates for 1–2 h at 4°C with a rotator. Protein A Sepharose beads (45-000-143; Thermo Fisher Scientific) were added to each IP for 1–2 h, again at 4°C with rotation, after which beads were spun down and washed three times with NP-40 lysis buffer. After the third spin, the wash was removed, and the beads were boiled in Laemmli buffer for 3 min at 95°C. The IP samples were loaded onto an 8% acrylamide gel along with 40 μl of the input samples. The proteins were transferred to a 0.45-µm nitrocellulose membrane and incubated at 4°C overnight in blocking buffer (5% BSA [wt/vol] dissolved in TBS-T [Tris-buffered saline with 0.1% Tween-20]). Membranes were incubated overnight at 4°C in primary antibody and washed three times in TBS-T and then at room temperature for 1 h with fluorescent secondary antibodies (LI-COR). Blots were imaged on an Odyssey CLX imager and then quantified in Image Studio Lite (LI-COR).

For IPs to detect O-GlcNAc moieties on GFP-TRAK1, 50 mM GlcNAc (A8625-5G; Sigma-Aldrich) and 2 µM ThiametG (110165CBC; MilliporeSigma) were also added to the IP buffer described above. IPs were performed as described above except the primary antibody used to immunoprecipitate GFP-TRAK was 3 µg of anti-TRAK1 (HPA005853; Sigma-Aldrich).

IPs from neurons: 5 × 10^6 neurons were lysed in 1 ml of the abovementioned NP-40 lysis buffer on ice. Lysates were centrifuged at 13,000 × *g* in a tabletop centrifuge for 10 min at 4°C. Supernatants were collected, 50 μl of which was boiled with SDS Laemmli buffer and used as input samples. The remaining supernatant was used for the IP. MYC-TRAK1, expressed from the human synapsin promoter (hSYN1), was immunoprecipitated with 10 μl MYC-Trap magnetic agarose beads for 2 h at 4°C. IP samples were washed three times with NP-40 lysis buffer and boiled in 30 μl of 1X Laemmli buffer. IP samples were run out on a 6% acrylamide gel and blotted as described above but using anti-HSP90 (60318-1-Ig; Proteintech) as a loading control.

### Western blotting

All cell types (neurons, fibroblasts, and HEKs) were lysed in NP-40 lysis buffer (see the Immunoprecipitations section for details) with the exception of the neuron lysates to detect ubiquitin and phospho-S65 ubiquitin, which were lysed as previously described (https://content.protocols.io/files/c39abixhp.pdf). SDS-PAGE was performed, and proteins were transferred and blocked as described above (see the Immunoprecipitations section). All blots were probed overnight at 4°C with the following antibody dilutions: rabbit anti-AMPK-α (1:1,000), rabbit anti-AMPK-α1 (1:1,000), rabbit anti-phospho-AMPK substrate motif (1:1,000), rabbit anti-phospho-AMPKα(T172) (1:1,000), rabbit anti-GAPDH (1:3,000), mouse anti-GAPDH (1:5,000), rabbit anti-GFP (1:1,000), mouse anti-HSP90 (1:5,000), mouse anti-MYC (9E10) (1:500), rabbit anti-phospho-ubiquitin (Ser65) (E2J6T) (1:1,000), mouse anti-O-linked N-acetylglucosamine (RL-2) (1:2,000), rabbit anti-tubulin (1:1,000), mouse anti-tubulin (DM1A) (1:2,000), mouse anti-vinculin (1:10,000), rabbit anti-vinculin (1:10,000), and mouse anti-ubiquitin (P4D1) (1:1,000). After the primary antibody incubation, blots were washed three times for 10 min in TBS-T and incubated for 1–2 h with the following LI-COR antibodies at a 1:5,000 dilution: anti-rabbit IRDye 800CW, anti-rabbit IRDye 680RD, anti-mouse IRDye 680RD, and anti-mouse IRDye 800CW. Blots were again washed three times for 10 min in TBS-T and imaged on an Odyssey CLX imager (LI-COR). All antibodies are listed in Table S3. Western blot images were quantified in Image Studio Lite (LI-COR). Quantifications were calculated using default Image Studio Light settings. Briefly, the pixel values of a region of interest (ROI) were first summed to general the “total” value. The product of the median background signal around the ROI and the ROI area were then subtracted from the total value to generate the “signal” value. Quantifications were normalized to a loading control to negate differences in lysate concentration and gel loading artifacts.

### LC-MS/MS sample preparation (in-gel digestion)

Using a light box to illuminate the gel, the protein bands were excised and cut into smaller pieces, then destained according to the vendor protocol (Pierce Silver Stain for Mass Spectrometry, Thermo Fisher Scientific), dehydrated by the addition of acetonitrile (ACN) (∼3 times the volume of gel pieces), and incubated at room temperature for 10 min. Gel pieces are shrunk and turn opaque, and ACN was removed. To reduce the cysteine residues, gel pieces were covered with a solution of freshly prepared 10 mM DTT in 50 mM ammonium bicarbonate (ABC) and agitated for 45 min at 55°C, and then, the reducing solution was removed. For the alkylation step, 55 mM acrylamide in 50 mM ABC was added and incubated at room temperature for 30 min. To terminate the alkylation, the solution was removed, and the gel pieces were washed twice under agitation for 10 min in 50 mM ABC followed by dehydration in ACN to turn opaque-white in color (∼5 min). Air-dried gel pieces were covered with 50 mM ABC containing 12.5 ng/µl trypsin (Promega) and allowed to swell for 60 min on ice. After rehydration, excess trypsin solution was removed, and gel pieces were covered with 50 mM ABC to ensure their immersion throughout digestion for 16 h at 37°C. The supernatant was transferred to a new tube, and the gel pieces were washed twice for 10 min with 300 μl of 2:1 (vol/vol) 50 mM ABC/ACN each and combined with the other supernatant. ACN was removed under reduced pressure, and samples were concentrated to ∼150 μl (Vacufuge plus, Eppendorf), acidified with formic acid (0.5%), desalted on NEST MicroSpin C18 spin columns (The Nest Group), concentrated to dryness using a speed vacuum (Vacufuge plus, Eppendorf), and resuspended in 5% formic acid/5% ACN loading buffer for LC-MS/MS.

### LC-MS/MS measurements

All samples were analyzed using trapped ion mobility spectrometry–quadrupole time-of-flight (timsTOF Pro) mass spectrometer coupled with ultra-high-pressure nano-flow liquid chromatography nanoElute system (Bruker). Peptides were loaded onto a reverse-phase 25-cm Aurora Series C18 analytical column (25 cm × 75 µm ID, 1.6 µm C18) fitted with a CaptiveSpray insert (IonOpticks). Column temperature was maintained at 50°C, and mobile phase A (2% ACN and 0.1% formic acid in water) and mobile phase B (0.1% formic acid in ACN) were used for the separation of peptides with 400 nl/min constant flow using a linear gradient starting from 0 to 30% in 90 min, followed by an increase to 80% B within 10 min, followed by washing and re-equilibration for 20 min. Mass spectra were acquired on a hybrid timsTOF Pro mass spectrometer with a modified nano-electrospray CaptiveSpray ion source (Bruker Daltonics). Mass spectrometer was operated in Parallel Accumulation SErial Fragmentation (PASEF) mode. Full mass spectra were acquired in a mass range of 100–1,700 m/z and ion mobility (1/k0) range from 0.60 to 1.60. 10 PASEF MS/MS scans per topN acquisition cycle were acquired.

### Analysis of MS data and identification of phosphorylation sites

The DDA timsTOF data were converted into MGF format using Bruker Data Analysis software. Files were searched using MASCOT Daemon search tool against the human UniProt reference proteome with digestion mode set to trypsin, fixed modification propionamide (C), and variable modifications acetyl (K), GG (K), oxidation (M), and phospho (ST). Files were also searched with MSFragger with the same search parameters, and all phosphorylation sites identified were compared. Average intensities of the phosphorylated peptides, normalized to total peptides of TRAK1, were converted to Log2, and the frequency of phosphorylation sites per group was calculated (see Table S2).

### Oxygen consumption (OCR)

E18 dissociated cortical neurons were plated on PDL-coated Seahorse 96-well cell culture dishes (see Cell culture methods) included in Seahorse XFe96 FluxPaks (102601-100; Agilent Technologies) at 5–7 × 10^4 cells/cm^2^. Flux packs were prepared according to the manufacturer’s protocol. One hour before the oxygen consumption measurements were made, 4 nM AntA (A8674; Sigma-Aldrich) or vehicle (i.e., dimethyl sulfoxide, D2650; Sigma-Aldrich) was added to neuronal Seahorse media (Ribeiro et al., 2014) and basal oxygen consumption was measured on a Seahorse XFe-96 analyzer. Immediately after Seahorse measurements were made, the number of nuclei per field was measured by fixing and Hoechst staining 5 wells each from AntA or vehicle-treated wells. Oxygen consumption was normalized to these counts.

### Statistical analysis

Plots were generated, and statistical analyses were performed using GraphPad Prism 10. All box and whiskers plots show the median value (middle bar) and the 25th and 75th quartiles (lower and upper bars of each box). The whiskers displayed are the 10th and 90th percentiles with the exception of Fig. S5 B, in which they show the minimum to maximum values. Ns are defined in each figure legend. While power analysis was not performed, sample sizes are similar to those commonly reported in the field. Formal testing for normally distributed data was not performed.

### Online supplemental material

Fig. S1 presents data in support of a model in which mitochondrial movement specifically, and not organelle movement in general, is decreased by 4 nM AntA treatment. Fig. S2 shows that the mitochondrial arrest caused by 4 nM AntA treatment is PINK1-independent. Fig. S3 provides controls for the rapalog system used in Fig. 2, as well as additional images and a montage of the perimitochondrial actin encasements described in Fig. 3. Fig. S4 demonstrates that Fhl2, Myo6, and PINK1 do not contribute to the arrest caused by 4 nM AntA. Fig. S5 displays kymographs and supporting data connecting AMPK to the AntA-induced mitochondrial arrest and the phosphorylation of TRAK1 (related to Figs. 5, 6, and 7). Table S1 lists primers used in this study; Table S2 lists phosphoproteomics data; and Table S3 lists plasmids, tool, and other reagents with their respective RRIDs.

## Supplementary Material

Table S1 lists primers used in this study.

Table S2 lists phosphoproteomics data.

Table S3 lists plasmids, tool, and other reagents with their respective RRIDs.

SourceData F4 is the source file for Fig. 4.

SourceData F5 is the source file for Fig. 5.

SourceData F6 is the source file for Fig. 6.

SourceData FS2 is the source file for Fig. S2.

SourceData FS5 is the source file for Fig. S5.

## Data Availability

The data are available from the corresponding author upon reasonable request.
