# Supplementary material for: Energy stress activates AMPK to arrest mitochondria via phosphorylation of TRAK1
Source: J Cell Biol. 2026 Jan 30;225(4):e202501023. doi: 10.1083/jcb.202501023 (PMC12857616; doi:10.1083/jcb.202501023)
Supplement: Table S3 — lists plasmids, tool, and other reagents with their respective RRIDs. [file jcb_202501023_tables3.docx]

**Table S3**

| **Reagents** | **Source** | **Catalog #, RRID and/or website** |  |
| --- | --- | --- | --- |
| **Antibodies** | | | |
| anti-AMPK-α (α1 ^70^and α2) | Cell Signaling | CAT#2532; RRID: AB_330331 |  |
| anti-AMPK-α1 | Cell Signaling | CAT#2795; RRID: AB_560856 |  |
| anti-Phospho AMPK Substrate Motif [LXRXX(pS/pT) | Cell Signaling | CAT#5759S; RRID: AB_10949320 |  |
| anti-Phospho-AMPKα (Thr172) (40H9) | Cell Signaling | CAT#2535S; RRID: AB_331250 |  |
| anti-GAPDH (14C10) | Cell Signaling | CAT#2118; RRID: AB_561053 |  |
| Anti-GADPH (6C5) | Millipore Sigma | CAT#AMAB91153; RRID: AB_2107426 |  |
| anti-GFP | ThermoFisher | CAT#A-11122; RRID: AB_221569 |  |
| CoraLite Plus 647-conjugated Anti HA Tag | Proteintech | CAT#CL647-66006; RRID: AB_3086564 |  |
| anti-HSP60 | Novus | CAT#NBP1-77397; RRID: AB_11034890 |  |
| anti-HSP90 | Proteintech | CAT#60318; RRID: AB_2881429 |  |
| anti-MYC (9E10) | Santa Cruz | CAT#SC-40; RRID: AB_627268 |  |
| anti-Phospho-Ubiquitin (Ser65) (E2J6T) | Cell Signaling | CAT#62802; RRID: AB_2799632 |  |
| anti-O-Linked N-Acetylglucosamine (RL-2) | Abcam | CAT#ab2739; RRID: AB_303264 |  |
| anti-TRAK1 | Millipore Sigma | CAT#HPA005853; RRID: AB_1854778 |  |
| anti-Tubulin | Millipore Sigma | CAT#SAB4500087; RRID: AB_10743646 |  |
| anti-Tubulin (DM1A) | Millipore Sigma | CAT#T6199; RRID: AB_477583 |  |
| anti-Vinculin | Proteintech | CAT#66305-1; RRID: AB_2810300 |  |
| anti-Vinculin | Proteintech | CAT#26520-1-AP; RRID: AB_2868558 |  |
| anti-Ubiquitin (P4D1) | Cell Signaling | CAT#3936; RRID: AB_331292 |  |
| anti-Rabbit IRDye 800CW | LiCor Biosciences | CAT#926-32213; RRID: AB_621848 |  |
| anti-Rabbit IRDye 680RD | LiCor Biosciences | CAT#926-68073; RRID: AB_10954442 |  |
| anti-Mouse IRDye 680RD | LiCor Biosciences | CAT#926-68072; RRID: AB_10953628 |  |
| anti-Mouse IRDye 800CW | LiCor Biosciences | CAT#926-32212; RRID: AB_621847 |  |
| anti-MYC (Myc-Trap Magnetic Agarose) | Proteintech | CAT#ytma; RRID: AB_2631370 |  |
| **Bacterial and virus strains** | | | |
| XL10 Gold *E. Coli* | Agilent | CAT#200314 |  |
| **Chemicals, peptides, and recombinant proteins** | | |  |
| AntimycinA | Sigma-Aldrich | A8674 |  |
| Oligomycin | Enzo Life Sciences | ALX-380-037-M010 |  |
| 2-Deoxy-D-glucose | Sigma-Aldrich | D8375-1GD8375 |  |
| Carbonyl cyanide m-chlorophenyl hydrazone (CCCP) | Cayman Chemical Company | 25458 |  |
| TMG (Thiamet-G) | EMDBiosciences | 110165CBC-25MG |  |
| Laemmli SDS sample buffer | ThermoFisher | J61337.AD |  |
| Phenylmethylsulfonyl Fluoride (PMSF) | EMD Millipore | 52332-1g |  |
| cOmplete Protease Inhibitor Cocktail | Sigma-Aldrich | 11836170001 |  |
| Phosstop-phosphatase inhibitor Cocktail | Sigma-Aldrich | 4906845001 |  |
| N-Acetyl-D-glucosamine | Sigma-Aldrich | A8625-5G |  |
| A/C Heterodimerizer (Rapalog) | Takara | 635056 |  |
| Dimethylsulfoxide (DMSO) | Sigma-Aldrich | D2650 |  |
| Image-iT™ TMRM Reagent | ThermoFisher | I34361 |  |
| Sucrose | Sigma-Aldrich | S0389 |  |
| Paraformaldehyde | Electron microscopy science | 15714 |  |
| Triton X-100 | Sigma-Aldrich | T8787-250ML |  |
| LysoTracker Red DND-99 | ThermoFisher | L7528 |  |
| Hoechst 33342 solution | ThermoFisher | 62249 |  |
| D-(+)-Galactose | Sigma-Aldrich | G5388-100G |  |
| D-(+)-Glucose | Sigma-Aldrich | D9434 |  |
| Sodium Pyruvate | Sigma-Aldrich | P8574 |  |
| Sodium L-lactate | Sigma-Aldrich | 71718 |  |
| Laminin | Life Technologies | 23017-015 |  |
| Poly-L-lysine hydrobromide | Sigma-Aldrich | P2636 |  |
| DMEM | Life Technologies | 10566-016 |  |
| DMEM without glucose | Life Technologies | 11-966-025 |  |
| Neurobasal+ | Life Technologies | A3582901 |  |
| Neurobasal A without glucose | Life Technologies | A2477501 |  |
| B27 Supplement | Life Technologies | A3582801 |  |
| Glutamax | Life Technologies | 35050061 |  |
| Penicillin-Streptomycin | Life Technologies | P4333 |  |
| Penicillin-Streptomycin-Glutamine | Life Technologies | 10378016 |  |
| Fetal Bovine Serum | Atlanta Biologicals | S11150H |  |
| Hibernate E | Fisher Scientific | NC0285514 |  |
| T4 Ligase | New England Biolabs | M0202S |  |
| AscI | New England Biolabs | R0558S |  |
| AgeI-HF | New England Biolabs | R3552L |  |
| NotI-HF | New England Biolabs | R0189S |  |
| BsrGI-HF | New England Biolabs | R3575S |  |
| MluI-HF | New England Biolabs | R3198S |  |
| BamHI-HF | New England Biolabs | R3136S |  |
| SpeI-HF | New England Biolabs | R3133S |  |
| MauBI | ThermoFisher | FD2084 |  |
| EcoRI-HF | New England Biolabs | R3101S |  |
| BglII | New England Biolabs | R0144S |  |
| PspoMI | New England Biolabs | R0653S |  |
| PEI Max | Polysciences | 24765-100 |  |
| Lipofectamine 2000 | ThermoFisher | 11668-019 |  |
| Seahorse FluxPaks | Agilent | 103793-100 |  |
| Seahorse XF Calibrant Solution | Agilent | 100840-000 |  |
| Seahorse XF DMEM Medium pH 7.4 | Agilent | 103575-100 |  |
| Gibson Assembly 2X Master Mix | New England Biolabs | E2611L |  |
| Q5® High-Fidelity 2X Master Mix | New England Biolabs | M0492L |  |
| NEB Site directed mutagenesis kit | New England Biolabs | E0554S |  |
| **Deposited data** | | | |
| MYC-hTRAK1 mass spec data | This paper | Table S2 |  |
| **Experimental models: Cell lines** | | |  |
| HEK293T/17 | ATCC | CAT#CRL-11268; RRID: CVCL_1926 |  |
| **Experimental models: Organisms/strains** | | |  |
| Neurons and Fibroblasts from Long Evans E18 Rats | Charles River Labs | CAT#006L/E; RRID: RGD_2308852 |  |
| Long EvansPINK1 Knockout Fibroblasts (HsdSage:LE-Pink1em1Sage) | Sage Labs (now Inotiv) | TGRL4690 |  |
| **Oligonucleotides** | | |  |
| See Table S1 |  |  |  |
| **Recombinant DNA** | | |  |
| pMD2.G | Gift from Didier Trono | RRID: Addgene_12259; JF440 |  |
| psPAX2 | Gift from Didier Trono | RRID: Addgene_12260; JF439 |  |
| pAMPK alpha2 K45R | (Mu et al., 2001) | RRID: Addgene_15992; JF451 |  |
| CMV-PercevalHR | (Werley et al., 2020) | RRID: Addgene_163061; JF639 |  |
| pLVX-CAG-mito-dsRED2 | This paper | RRID: Addgene_173071; JF264 |  |
| iRFP-Trim46 | This paper | RRID: Addgene_176266; JF292 |  |
| GFP-hTrak1 | This paper | RRID: Addgene_188664; JF474 |  |
| 2X-FKBP-meGFP-OMP25mts | This paper | RRID: Addgene_219567; JF188 |  |
| pLVX-EF1a-mito-mRaspberry | This paper | RRID: Addgene_219568; JF536 |  |
| pLVX-CAG-Ftractin-GFP | This paper | RRID: Addgene_219569; JF529 |  |
| MYC-hTRAK1(S919A) | This paper | RRID: Addgene_219570; JF543 |  |
| MYC-hTRAK1(S719A) | This paper | RRID: Addgene_219571; JF557 |  |
| MYC-hTRAK1(S200A, S201A) | This paper | RRID: Addgene_219572; JF559 |  |
| MYC-hTRAK1(T834A) | This paper | RRID: Addgene_219573; JF571 |  |
| MYC-hTRAK1(S200A, S201A, S719A, T834A, S919A) | This paper | RRID: Addgene_219574; JF579 |  |
| pLVX-CAG-meGFP-Myo6 | This paper | RRID: Addgene_219575; JF619 |  |
| pLVX-CAG-Fhl2-HA-P2A-mitomeGFP | This paper | RRID: Addgene_219576; JF626 |  |
| pLVX-hSYN-MYC-MIRO1 | This paper | RRID: Addgene_219577; JF525 |  |
| MYC-hTRAK2 | (Davis et al., 2022) | RRID: Addgene_225142; JF345 |  |
| hSYN-meGFP | This paper | RRID: Addgene_225201; JF651 |  |
| MYC-hTRAK1 | (Pekkurnaz et al., 2014) | RRID: Addgene_225204; JF190 |  |
| pCherry-FRB | (Karginov et al., 2010) | RRID: Addgene_25920; JF196 |  |
| pEBG‐AMPK α1(1‐312) | (Egan et al., 2011) | RRID: Addgene_27632; JF450 |  |
| FUGW-PercevalHR | (M. Tantama et al., 2013) | RRID: Addgene_49083; JF629 |  |
| mEmerald-Rab5a-7 | Gift from Michael Davidson | RRID: Addgene_54243; JF124 |  |
| mEGFP-N1 | Gift from Michael Davidson | RRID: Addgene_54767; JF31 |  |
| HA-Kif5b-MD-FRB | (Kapitein et al., 2010) | JF184 |  |
| nGFP-OGT-4 | (Ramirez et al., 2020) | JF426 |  |
| (294) pcdna3.1-myc-OGA(1-400) | (Ge et al., 2021) | JF423 |  |
| (369) pcdna3.1-HA-nLaGG-OGA-(544-706) (nGFP-C3) | (Ge et al., 2021) | JF425 |  |
| LVX-hSYN1-mNeonGreen | Gift from Isaac Chiu's lab | JF517 |  |
| shRNA AMPKa1 NLS-iRFP | Sigma | Sigma mission shRNA targeting mouse AMPKα1 (TRCN0000360842) in which the puromycin resistance cassette was replaced with NLS-iRFP702; JF587 |  |
| Control shRNA2 NLS-iRFP | Sigma | Sigma mission non-targeting control shRNA (SHC216) in which the puromycin resistance cassette was replaced with NLS-iRFP702; JF585 |  |
| pLVX-hSYN-mito-dsRED2 | This paper | RRID: Addgene_173069; JF276 |  |
| pLVX-EF1a-mito-dsRED2 | This paper | RRID: Addgene_174541; JF277 |  |
| GFP-HALO-OMP25 | This paper | RRID: Addgene_188661; JF20 |  |
| pchBA-meGFP-HALO-OMP25(MTS) | This paper | RRID: Addgene_188663; JF61 |  |
| pLVX-CAG-MCS | This paper | RRID: Addgene_225209; JF580 |  |
| hTRAK1(S200A, S201A, S719A, S919A) | This paper | RRID: Addgene_225225; JF565 |  |
| hTRAK1(S719A, S919A) | This paper | RRID: Addgene_225226; JF560 |  |
| pCMV-meGFP-Myo6 | This paper | RRID: Addgene_225227; JF576 |  |
| TOMM20(MTS)-HaloTag-mEGFP | This paper | RRID: Addgene_225267; JF51 |  |
| pCMV-MCS-P2A-mCherry-N1 | This paper | RRID: Addgene_225268; JF128 |  |
| Fhl2-HA-P2A-meGFP(omp25mts) | This paper | RRID: Addgene_225269; JF570 |  |
| pCMV-MCS-P2A-meGFP(omp25mts) | This paper | RRID: Addgene_225270; JF146 |  |
| AmCyan-P2A-mCherry | (Potorac et al., 2016) | RRID: Addgene_45350; JF75 |  |
| mRaspberry-Mito-7 | Gift from Michael Davidson | RRID: Addgene_55931; JF46 |  |
| pEGFP-C1 F-tractin-EGFP | (Belin et al., 2014) | RRID: Addgene_58473; JF258 |  |
| pbetaActin-HALO-GFP-preActA (pERB254) | (Ballister et al., 2015) | RRID: Addgene_67762; JF11 |  |
| paGFP-OMP25 | (Katajisto et al., 2015) | RRID: Addgene_69598; JF19 |  |
| pExpress-MYO6 | Transomic | CAT#BC146764.1; JF155 |  |
| PINK1-D110-YFP-FKBP | (Lazarou et al., 2012) | JF6 |  |
| MYC-Miro1 | (Fransson et al., 2006) | RRID: Addgene_225204; JF151 |  |
| pLVX-CAG-MCS-IRES-mCherry | Gift from Matt Lavoie | JF250 |  |
| pLV-EF1a-IRES-Hygro | (Hayer et al., 2016) | RRID: Addgene_85134; JF276 |  |
| pLV-hSYN-RFP | (Nathanson et al., 2009) | RRID: Addgene_22909; JF252 |  |
| DsRed2-Mito-7 | Gift from Michael Davidson | RRID: Addgene_55838; JF262 |  |
| piRFP702-N1 | (Shcherbakova and Verkhusha, 2013) | RRID: Addgene_45456; JF105 |  |
| DsRed2-N1 | Gift from Michael Davidson | RRID: Addgene_54493; JF30 |  |
| pERB254 pbetaActin-HALO-GFP-preActA | (Ballister et al., 2015) | RRID: Addgene_67762; JF11 |  |
| meGFP-C1 | Gift from Michael Davidson | RRID: Addgene_54759; JF154 |  |
| pLVX-CAG-mito-dsRED2 | This paper | RRID: Addgene_173071; JF264 |  |
| iRFP-Trim46 | This paper | RRID: Addgene_176266; JF292 |  |
| meGFP-hTrak1 | This paper | RRID: Addgene_188664; JF474 |  |
| mCherry-Trim46 | (Van Beuningen et al., 2015) | RRID: Addgene_176401; JF236 |  |
| pLVX-EF1a-mito-dsRED2 | This paper | RRID: Addgene_174541; JF277 |  |
| meGFP-N1 | Gift from Michael Davidson | RRID: Addgene_54767; JF154 |  |
| GFP-HALO-OMP25 | This paper | RRID: Addgene_226880; JF20 |  |
| pchBA-meGFP-HALO-OMP25(MTS) | This paper | RRID: Addgene_226881; JF61 |  |
| pTomm20-mCherry-FKBP | A gift from Takanari Inoue | JF41 |  |
| pCMV- TOMM20(MTS)-Halo-dsRED2 | This paper | RRID: Addgene_226883; JF47 |  |
| FHL2-HA-OMP25 | (Basu et al., 2021) | JF349 |  |
| pCMV-HALO-dsRED2-N1 | This paper | RRID: Addgene_226882_; JF39 |  |
| shRNA TRAK1 NLS-iRFP | Sigma | Sigma mission shRNA to human TRAK1 (TRCN0000036275) in which the puromycin resistance cassette was replaced with NLS-iRFP702; JF684 |  |
| Control shRNA1 NLS-iRFP | Sigma | Sigma mission non-targeting control shRNA (SHC016) in which the puromycin resistance cassette was replaced with NLS-iRFP702; JF306 |  |
| Control shRNA1 | Sigma | Sigma mission non-targeting control shRNA (SHC016); JF159 |  |
| shRNA Fhl2 | Sigma | Sigma mission shRNA to human Fhl2 TCRN0000005773; JF165 |  |
| **Software and algorithms** | | |  |
| Fiji | (Schindelin et al., 2012) | RRID: SCR_001935 |  |
| Kymolyzer (Fiji plugin) | (Basu et al., 2020) | N/A |  |
| QuovadoPro (Fiji plugin) | (Basu and Schwarz, 2020) | N/A |  |
| Image Studio Lite | LICORbio | <https://www.licor.com/bio/image-studio-lite/> |  |
| TrackMate7 | (Ershov et al., 2022) | N/A |  |
| Word and Excel (Microsoft Office 365) | Microsoft | <https://www.microsoft.com/> |  |
| Adobe Illustrator | Adobe | <https://www.adobe.com/> |  |
| Prism 10 | Dotmatics | <https://www.graphpad.com/features> |  |

**References**

Ballister, E.R., S. Ayloo, D.M. Chenoweth, M.A. Lampson, and E.L.F. Holzbaur. 2015. Optogenetic control of organelle transport using a photocaged chemical inducer of dimerization. *Current Biology*. 25:R407–R408. doi:10.1016/j.cub.2015.03.056.

Basu, H., L. Ding, G. Pekkurnaz, M. Cronin, and T.L. Schwarz. 2020. Kymolyzer, a Semi-Autonomous Kymography Tool to Analyze Intracellular Motility. *Curr Protoc Cell Biol*. 87. doi:10.1002/cpcb.107.

Basu, H., G. Pekkurnaz, J. Falk, W. Wei, M. Chin, J. Steen, and T.L. Schwarz. 2021. Fhl2 anchors mitochondria to actin and adapts mitochondrial dynamics to glucose supply. *Journal of Cell Biology*. 220. doi:10.1083/jcb.201912077.

Basu, H., and T.L. Schwarz. 2020. QuoVadoPro, an Autonomous Tool for Measuring Intracellular Dynamics using Temporal Variance. *Curr Protoc Cell Biol*. 87:e108. doi:10.1002/cpcb.108.

Belin, B.J., L.M. Goins, and R.D. Mullins. 2014. Comparative analysis of tools for live cell imaging of actin network architecture. *Bioarchitecture*. 4:189–202. doi:10.1080/19490992.2014.1047714.

Van Beuningen, S.F.B., L. Will, M. Harterink, A. Chazeau, E.Y. Van Battum, C.P. Frias, M.A.M. Franker, E.A. Katrukha, R. Stucchi, K. Vocking, A.T. Antunes, L. Slenders, S. Doulkeridou, P. Sillevis Smitt, A.F.M. Altelaar, J.A. Post, A. Akhmanova, R.J. Pasterkamp, L.C. Kapitein, E. de Graaff, and C.C. Hoogenraad. 2015. TRIM46 Controls Neuronal Polarity and Axon Specification by Driving the Formation of Parallel Microtubule Arrays. *Neuron*. 88:1208–1226. doi:10.1016/j.neuron.2015.11.012.

Davis, K., H. Basu, I. Izquierdo-Villalba, E. Shurberg, and T.L. Schwarz. 2022. Miro GTPase domains regulate the assembly of the mitochondrial motor-adaptor complex. *Life Sci Alliance*. 6. doi:10.26508/lsa.202201406.

Egan, D.F., D.B. Shackelford, M.M. Mihaylova, S. Gelino, R.A. Kohnz, W. Mair, D.S. Vasquez, A. Joshi, D.M. Gwinn, R. Taylor, J.M. Asara, J. Fitzpatrick, A. Dillin, B. Viollet, M. Kundu, M. Hansen, and R.J. Shaw. 2011. Phosphorylation of ULK1 (hATG1) by AMP-activated protein kinase connects energy sensing to mitophagy. *Science (1979)*. 331:456–461. doi:10.1126/science.1196371.

Ershov, D., M.S. Phan, J.W. Pylvänäinen, S.U. Rigaud, L. Le Blanc, A. Charles-Orszag, J.R.W. Conway, R.F. Laine, N.H. Roy, D. Bonazzi, G. Duménil, G. Jacquemet, and J.Y. Tinevez. 2022. TrackMate 7: integrating state-of-the-art segmentation algorithms into tracking pipelines. *Nat Methods*. 19:829–832. doi:10.1038/s41592-022-01507-1.

Fransson, Å., A. Ruusala, and P. Aspenström. 2006. The atypical Rho GTPases Miro-1 and Miro-2 have essential roles in mitochondrial trafficking. *Biochem Biophys Res Commun*. 344:500–510. doi:10.1016/j.bbrc.2006.03.163.

Ge, Y., D.H. Ramirez, B. Yang, A.K. D’Souza, C. Aonbangkhen, S. Wong, and C.M. Woo. 2021. Target protein deglycosylation in living cells by a nanobody-fused split O-GlcNAcase. *Nat Chem Biol*. 17. doi:10.1038/s41589-021-00757-y.

Hayer, A., L. Shao, M. Chung, L.M. Joubert, H.W. Yang, F.C. Tsai, A. Bisaria, E. Betzig, and T. Meyer. 2016. Engulfed cadherin fingers are polarized junctional structures between collectively migrating endothelial cells. *Nat Cell Biol*. 18:1311–1323. doi:10.1038/ncb3438.

Kapitein, L.C., M.A. Schlager, W.A. Van Der Zwan, P.S. Wulf, N. Keijzer, and C.C. Hoogenraad. 2010. Probing intracellular motor protein activity using an inducible cargo trafficking assay. *Biophys J*. 99:2143–2152. doi:10.1016/j.bpj.2010.07.055.

Karginov, A. V., F. Ding, P. Kota, N. V. Dokholyan, and K.M. Hahn. 2010. Engineered allosteric activation of kinases in living cells. *Nat Biotechnol*. 28:743–747. doi:10.1038/nbt.1639.

Katajisto, P., J. Döhla, C.L. Chaffer, N. Pentinmikko, N. Marjanovic, S. Iqbal, R. Zoncu, W. Chen, R.A. Weinberg, and D.M. Sabatini. 2015. Asymmetric apportioning of aged mitochondria between daughter cells is required for stemness. *Science (1979)*. 348:340–343. doi:10.1126/science.1260384.

Lazarou, M., S.M. Jin, L.A. Kane, and R.J. Youle. 2012. Role of PINK1 Binding to the TOM Complex and Alternate Intracellular Membranes in Recruitment and Activation of the E3 Ligase Parkin. *Dev Cell*. 22:320–333. doi:10.1016/j.devcel.2011.12.014.

M. Tantama, M.-F. JR, M. R, and G Yellen. 2013. Imaging energy status in live cells with a fluorescent biosensor of the intracellular ATP-to-ADP ratio. *Nat Commun*. 4. doi:https://doi.org/10.1038/ncomms3550.

Mu, J., J.T. Brozinick, O. Valladares, M. Bucan, and M.J. Birnbaum. 2001. A Role for AMP-Activated Protein Kinase in Contraction-and Hypoxia-Regulated Glucose Transport in Skeletal Muscle hexose utilization in skeletal muscle. *Mol Cell*. 7:1085–1094.

Nathanson, J.L., Y. Yanagawa, K. Obata, and E.M. Callaway. 2009. Preferential labeling of inhibitory and excitatory cortical neurons by endogenous tropism of adeno-associated virus and lentivirus vectors. *Neuroscience*. 161:441–450. doi:10.1016/j.neuroscience.2009.03.032.

Pekkurnaz, G., J.C. Trinidad, X. Wang, D. Kong, and T.L. Schwarz. 2014. Glucose regulates mitochondrial motility via Milton modification by O-GlcNAc transferase. *Cell*. 158:54–68.

Potorac, I., A. Rivero-Müller, A. Trehan, M. Kielbus, K. Jozwiak, F. Pralong, A. Hafidi, A. Thiry, J.J. Ménagé, I. Huhtaniemi, A. Beckers, and A.F. Daly. 2016. A vital region for human glycoprotein hormone trafficking revealed by an LHB mutation. *Journal of Endocrinology*. 231:197–207. doi:10.1530/JOE-16-0384.

Ramirez, D.H., C. Aonbangkhen, H.Y. Wu, J.A. Naftaly, S. Tang, T.R. O’Meara, and C.M. Woo. 2020. Engineering a Proximity-Directed O-GlcNAc Transferase for Selective Protein O-GlcNAcylation in Cells. *ACS Chem Biol*. 15:1059–1066. doi:10.1021/acschembio.0c00074.

Schindelin, J., I. Arganda-Carreras, E. Frise, V. Kaynig, M. Longair, T. Pietzsch, S. Preibisch, C. Rueden, S. Saalfeld, B. Schmid, J.Y. Tinevez, D.J. White, V. Hartenstein, K. Eliceiri, P. Tomancak, and A. Cardona. 2012. Fiji: An open-source platform for biological-image analysis. *Nat Methods*. 9:676–682. doi:10.1038/nmeth.2019.

Shcherbakova, D.M., and V. V. Verkhusha. 2013. Near-infrared fluorescent proteins for multicolor in vivo imaging. *Nat Methods*. 10:751–754. doi:10.1038/nmeth.2521.

Werley, C.A., S. Boccardo, A. Rigamonti, E.M. Hansson, and A.E. Cohen. 2020. Multiplexed Optical Sensors in Arrayed Islands of Cells for multimodal recordings of cellular physiology. *Nat Commun*. 11. doi:10.1038/s41467-020-17607-5.
