# Supplementary figures and images for: Energy stress activates AMPK to arrest mitochondria via phosphorylation of TRAK1
Source: J Cell Biol. 2026 Jan 30;225(4):e202501023. doi: 10.1083/jcb.202501023 (PMC12857616; doi:10.1083/jcb.202501023)

Scanned at 700 (LiCor)

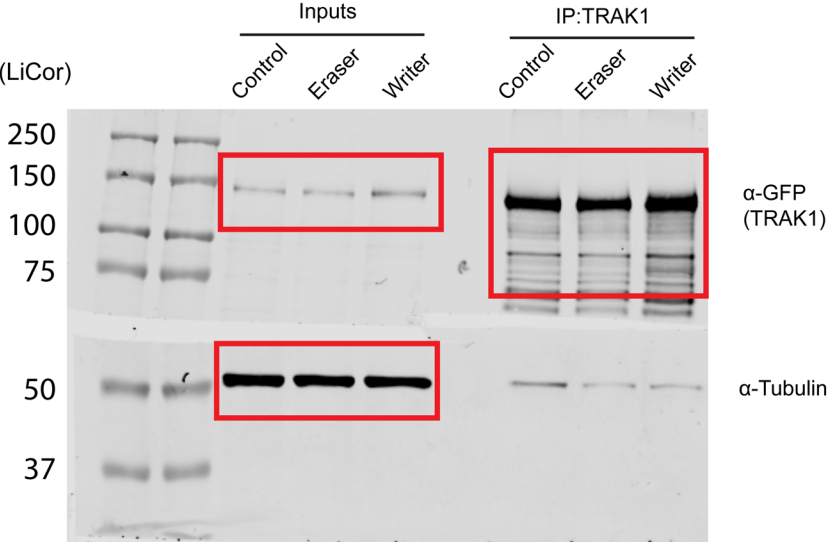

Scanned at 800 (LiCor)

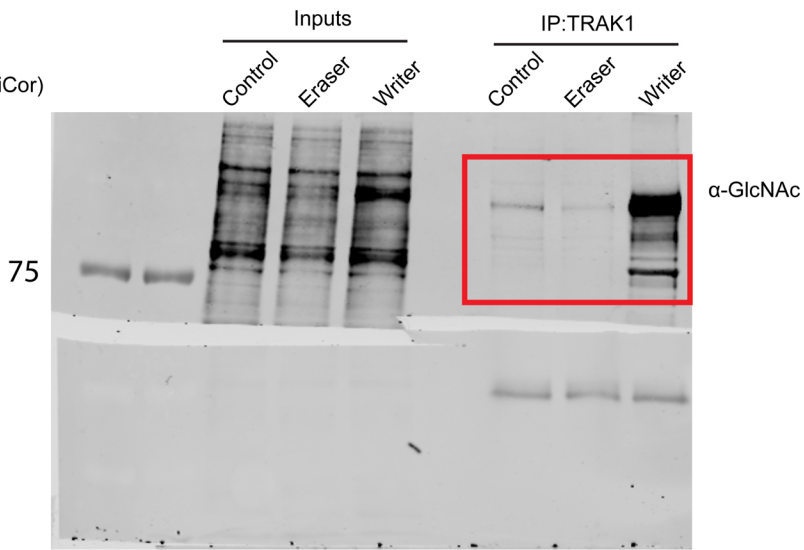

Supplement: SourceData F4 — is the source file for Fig. 4. [file jcb_202501023_sourcedataf4.pdf]

Scanned at 700 (LiCor)

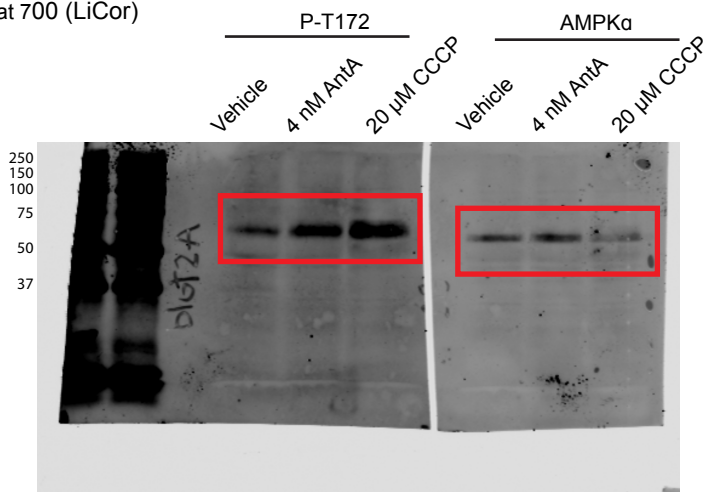

Scanned at 800 (LiCor)

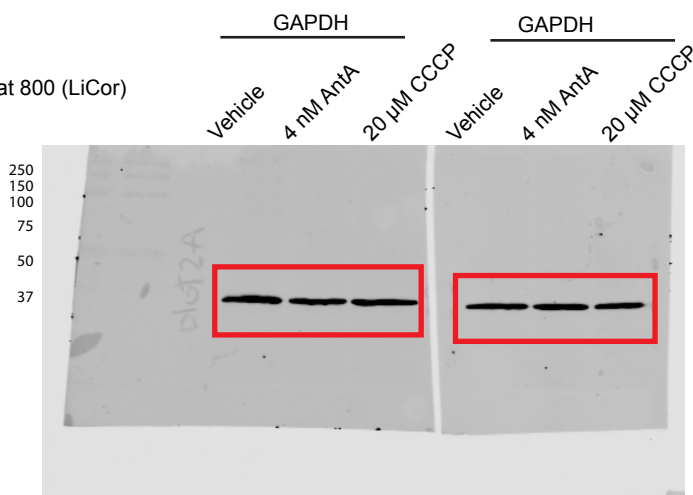

Supplement: SourceData F5 — is the source file for Fig. 5. [file jcb_202501023_sourcedataf5.pdf]

**A**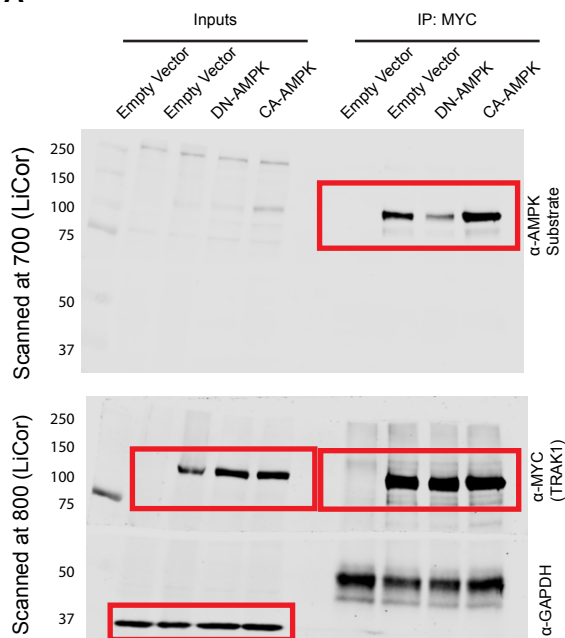**G**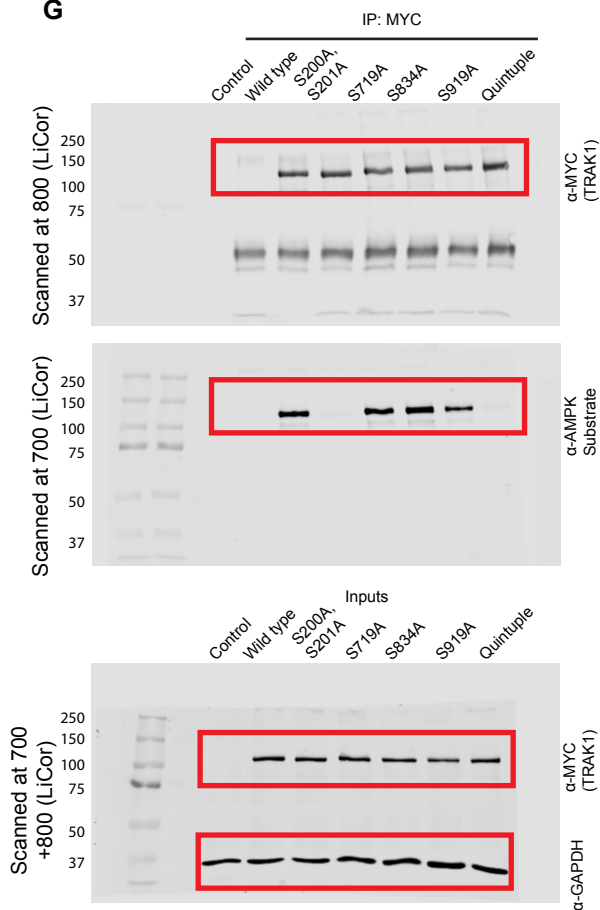**D**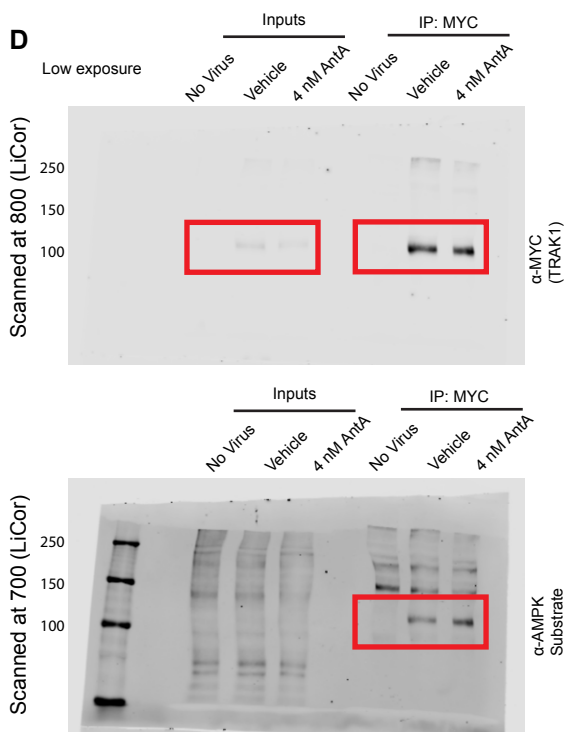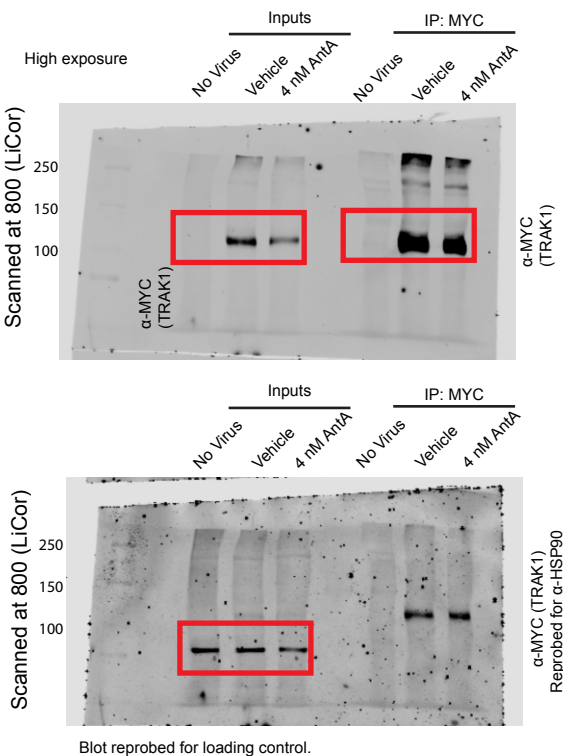

Supplement: SourceData F6 — is the source file for Fig. 6. [file jcb_202501023_sourcedataf6.pdf]

**A**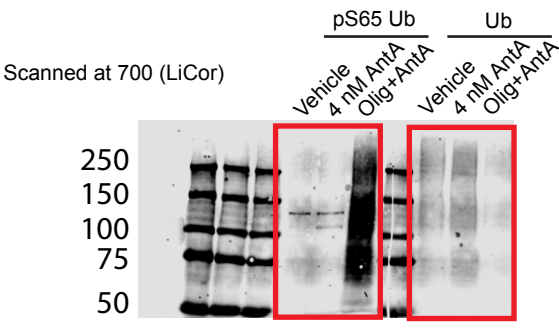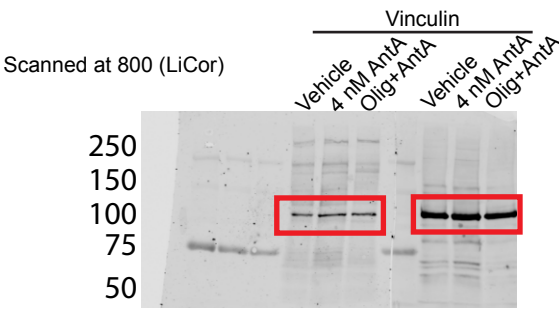**G**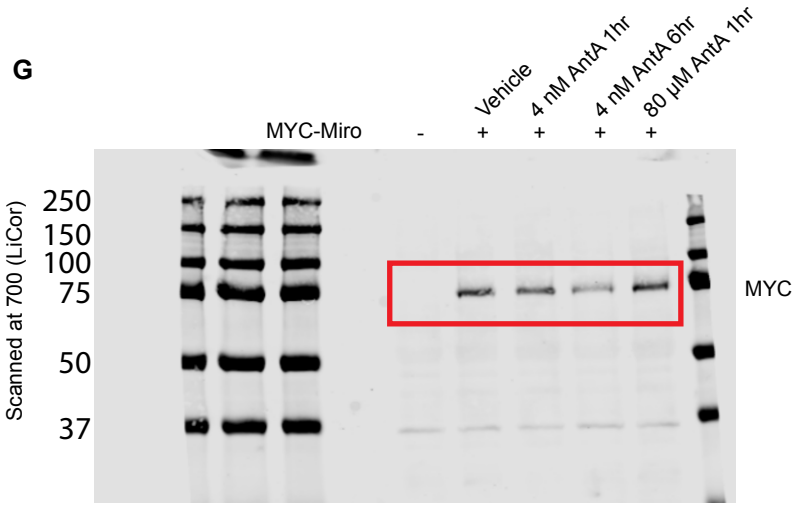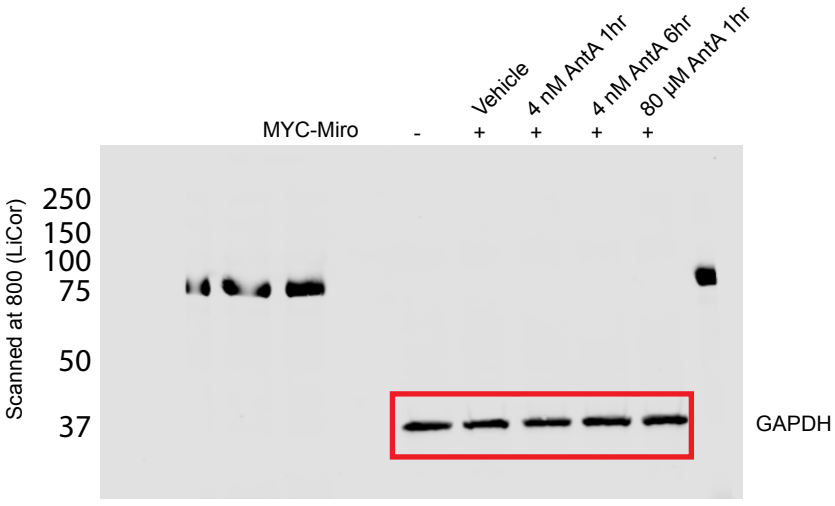

Supplement: SourceData FS2 — is the source file for Fig. S2. [file jcb_202501023_sourcedatafs2.pdf]
