# Supplementary material for: Energy stress activates AMPK to arrest mitochondria via phosphorylation of TRAK1
Source: J Cell Biol. 2026 Jan 30;225(4):e202501023. doi: 10.1083/jcb.202501023 (PMC12857616; doi:10.1083/jcb.202501023)
Supplement: SourceData FS5 — is the source file for Fig. S5. [file jcb_202501023_sourcedatafs5.pdf]

D

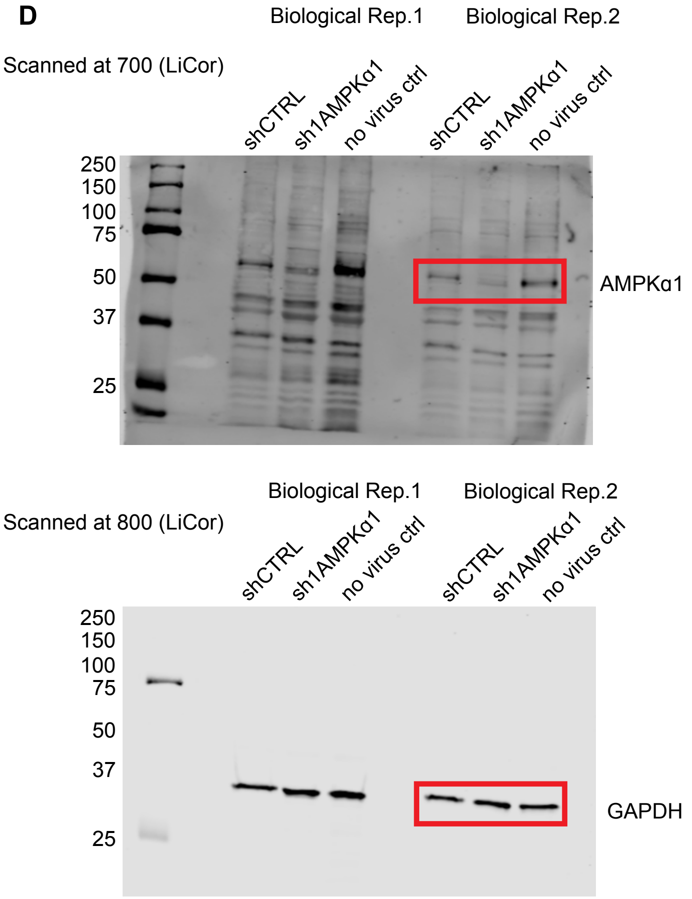
Biological Rep.1
Biological Rep.2
Scanned at 700 (LiCor)
shCTRL
sh1AMPKα1
no virus ctrl
shCTRL
sh1AMPKα1
no virus ctrl
250
150
100
75
50
37
25
AMPKα1
Biological Rep.1
Biological Rep.2
Scanned at 800 (LiCor)
shCTRL
sh1AMPKα1
no virus ctrl
shCTRL
sh1AMPKα1
no virus ctrl
250
150
100
75
50
37
25
GAPDH

G

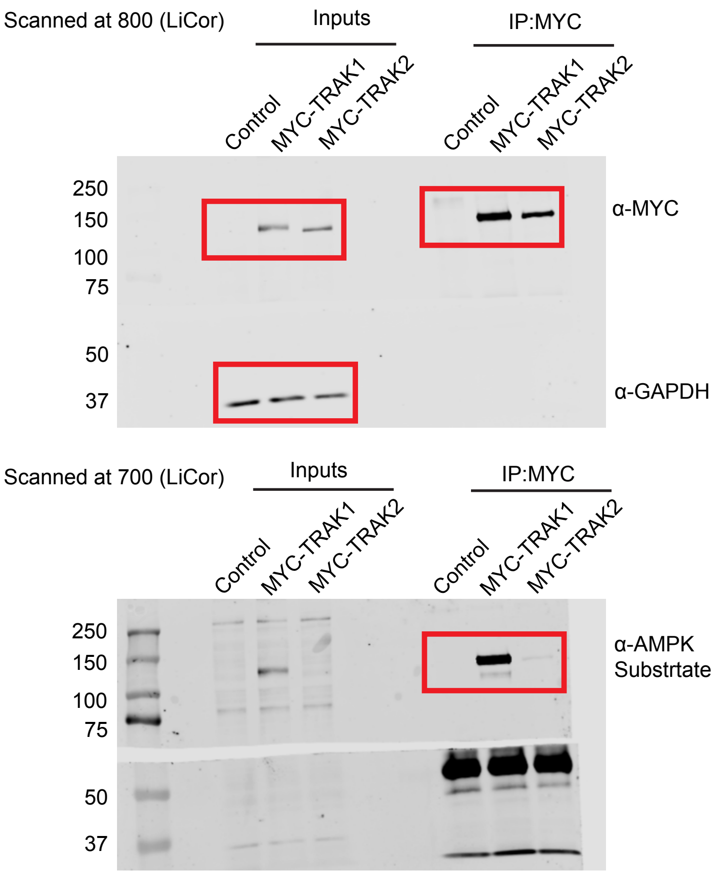
Scanned at 800 (LiCor)
Inputs
IP:MYC
Control
MYC-TRAK1
MYC-TRAK2
Control
MYC-TRAK1
MYC-TRAK2
250
150
100
75
50
37
α-MYC
α-GAPDH
Scanned at 700 (LiCor)
Inputs
IP:MYC
Control
MYC-TRAK1
MYC-TRAK2
Control
MYC-TRAK1
MYC-TRAK2
250
150
100
75
50
37
α-AMPK
Substrtate
